# Osteochondrogenesis by TGF-β3, BMP-2 and noggin growth factor combinations in an *ex vivo* muscle tissue model: Temporal function changes affecting tissue morphogenesis

**DOI:** 10.3389/fbioe.2023.1140118

**Published:** 2023-03-16

**Authors:** Heng Liu, Peter E. Müller, Attila Aszódi, Roland M. Klar

**Affiliations:** ^1^ Department of Orthopaedics and Trauma Surgery, Musculoskeletal University Center Munich (MUM), University Hospital, LMU Munich, Munich, Germany; ^2^ Department of Orthopaedics and Traumatology, Beijing Jishuitan Hospital, The Fourth Medical College of Peking University, Beijing, China; ^3^ Department of Oral and Craniofacial Sciences, University of Missouri-Kansas City, Kansas City, MO, United States

**Keywords:** tissue engineering, TGF-β3, BMP-2, noggin, temporal modulation, muscle tissue

## Abstract

In the absence of clear molecular insight, the biological mechanism behind the use of growth factors applied in osteochondral regeneration is still unresolved. The present study aimed to resolve whether multiple growth factors applied to muscle tissue *in vitro*, such as TGF-β3, BMP-2 and Noggin, can lead to appropriate tissue morphogenesis with a specific osteochondrogenic nature, thereby revealing the underlying molecular interaction mechanisms during the differentiation process. Interestingly, although the results showed the typical modulatory effect of BMP-2 and TGF-β3 on the osteochondral process, and Noggin seemingly downregulated specific signals such as BMP-2 activity, we also discovered a synergistic effect between TGF-β3 and Noggin that positively influenced tissue morphogenesis. Noggin was observed to upregulate BMP-2 and OCN at specific time windows of culture in the presence of TGF-β3, suggesting a temporal time switch causing functional changes in the signaling protein. This implies that signals change their functions throughout the process of new tissue formation, which may depend on the presence or absence of specific singular or multiple signaling cues. If this is the case, the signaling cascade is far more intricate and complex than originally believed, warranting intensive future investigations so that regenerative therapies of a critical clinical nature can function properly.

## 1 Introduction

Successful regeneration of cartilage and bone remains an unresolved enigma to be solved clinically (Pittenger et al., 2019; Xiong et al., 2021). Due to intrinsic limitations in the ability of articular cartilage to self-renew and repair, cartilage-related injuries often result in osteoarthritic degeneration and long-term pain (Huang et al., 2019; Huynh et al., 2019). Among numerous restoration techniques, osteochondral grafts hold a more favorable prognosis than cartilage grafts alone because the bone-to-bone interface is more likely to integrate than the cartilage-to-chondral interface (Schaefer et al., 2002; Sheehy et al., 2013). An engineered osteochondral construct with cartilage and bone phenotypes seems to be a potential strategy for the treatment of chondral and osteochondral defects (Schaefer et al., 2002; Alhadlaq and Mao, 2005). During the past decade, although some great successes have been achieved to engineer ideal biomimetic osteochondral tissue, numerous challenges still need to be cleared to realize its final clinical application (Chen et al., 2011; Rodrigues et al., 2012; Zhang et al., 2019). Therefore, alternative models and improved osteochondral tissue engineering (TE) technologies should be explored.

According to previous studies, the growth factors-loaded, muscle tissue-based, biomaterial induction system is a promising novel technology for TE (Betz et al., 2015; Betz et al., 2018; Ren et al., 2018; Xiong et al., 2020). Muscle is a relatively easily obtained tissue with a firm and durable self-repair capability; thus, harvesting muscle tissue does not cause severe morbidity in the donor area (Betz et al., 2009). It is well known that muscle tissue is an attractive cell source for TE since it contains abundant stem cells, which possess the potential to differentiate into an osteogenic lineage (Bosch et al., 2000; Ren et al., 2019). Compared to traditional cell culture-based TE approaches, the tissue culture system does not require the extraction and proliferation of autologous-derived osteoprogenitor cells, thus making it easier to operate and much cheaper (Betz et al., 2008; Virk et al., 2011). Additionally, the muscle tissue fragment is a one hundred percent biocompatible scaffold with a complex three-dimensional (3D) structure (Betz et al., 2008; Ren et al., 2019). Its intrinsic extracellular matrix (ECM) contains the necessary amino acids and the essential signaling molecules, providing an in vivo-like culturing milieu that supports cell growth and activity (Brand, 1997; Albert, 2005; Blair et al., 2017). Moreover, as a natural soft tissue scaffold, its easy deformability facilitates its matching to osteochondral defect sites. Furthermore, muscle tissue typically contains tiny blood vessels and numerous capillaries critical for nutrient flow and anabolic activities (Betz et al., 2013; Perniconi and Coletti, 2014; He et al., 2020).

Members of the transforming growth factor-beta (TGF-β) superfamily perform various pleiotropic functions during both antenatal and postnatal development (Alliston et al., 2008). Among them, TGF-β3 and bone morphogenetic protein-2 (BMP-2) play crucial roles in processes of skeletogenesis, including the regulation of mesenchymal stem cell condensation, chondrocyte and osteoblast differentiation, and growth plate expansion (Ripamonti et al., 2016; Wu et al., 2016). TGF-β3 has a bi-functional impact on the maintenance of cartilage metabolic homeostasis, as it favors early-stage chondrocyte proliferation but arrests downstream chondrocyte hypertrophy, which is crucial to preserving hyaline cartilage integrity (Kato et al., 1988; Wu et al., 2016). However, TGF-β signaling is also known to induce osteogenesis and accelerate osteoarthritis through a Smad2/3 independent pathway (van der Kraan et al., 2012; van der Kraan, 2014). The osteogenic potential of TGF-β3 has been demonstrated in many different models. For instance, Ripamonti et al. (2015) identified in vivo experiments that TGF-β3 functions as the crucial signaling in regulating osteogenic relative gene expression and thus inducing ectopic bone formation in baboons. BMP-2 is a prerogative molecule during bone formation, as it plays a role in nearly the entire endochondral bone formation process (Gazzerro and Canalis, 2006; Ripamonti, 2006). Evidence has shown that BMP-2 is one of the most potent inducers for osteogenic differentiation (Huang et al., 2010), in which Noel et al. (2004) certified that even a short duration of BMP-2 expression is sufficient to induce irreversible endochondral bone. Moreover, amongst its other tissue-inductive capabilities, BMP-2 can also promote chondrogenesis (Keller et al., 2011; Chen et al., 2020). The first evidence of this ability was given by Urist (1965), who discovered that BMP-2 could induce both ectopic cartilage and bone formation within the rectus abdominis muscle of adult rabbits.

As a classical extracellular antagonist of BMP-2, Noggin performs pleiotropic roles in various physiological and pathological developmental processes, such as the induction of neural and skeletal muscle tissue in early embryogenesis (Smith and Harland, 1992), and it is also crucial for chondrogenic and osteogenic differentiation (Bayramov et al., 2011; Krause et al., 2011). In mice overexpressing Noggin in the skeleton, osteoblast differentiation and bone formation were impaired, resulting in decreased bone mineral density and weakened osteoblastic function (Devlin et al., 2003; Wu et al., 2003). Nevertheless, the downregulation of Noggin in cells in the bone environment increases the expression of osteogenic differentiation markers and thus enhances the regeneration of bone defects (Gazzerro et al., 2003; Wan et al., 2007). Furthermore, proximal symphalangism and multiple synostoses syndrome in humans can also be attributed to Noggin mutations (Gong et al., 1999).

Previous experimental studies have reported that a combination of morphogens acting synergistically or in modulatory roles could result in superior morphogenesis (Cicione et al., 2015; Huang et al., 2020). For example, Xiong et al. (2020) demonstrated that the combined treatment of TGF-β3, BMP-2, and BMP-7 could promote chondrogenesis in muscle tissue more efficiently than either morphogen applied on its own or in various duplicate combinations. Similar synergistic effects have also been investigated by other scientists, in which co-administration of BMP-2 and TGF-β3 resulted in an improved bone formation response (Haschtmann et al., 2012; Wang et al., 2016; He et al., 2019). However, the antagonistic effect between different TGF-βs and BMPs has also been discussed by other researchers (Mehlhorn et al., 2007; Wakefield and Hill, 2013; Xiong, 2020). In addition, the mutual impact between BMPs and Noggin has been intensively explored in the last decades (Re’em-Kalma et al., 1995; Zakin and De Robertis, 2010; Wang et al., 2013). Recent studies have also shown the association between TGF-β3 and Noggin during the process of endochondral bone formation within muscle tissue (Klar et al., 2014; Ripamonti et al., 2015). Nevertheless, the detailed complex interaction mechanisms among these three growth factors and their temporal and spatial behavior have yet to be thoroughly explained.

Therefore, the present study attempted to detect what the osteochondrogenic effects, if any, would be under a temporal signaling cascade of these three growth factors, which are applied to this specialized muscle tissue model platform in seven different patterns. The differentiated cultured muscle tissue was analyzed at 7, 14, and 30 days using three methods (Pittenger et al., 2019): quantitative reverse transcription-polymerase chain reaction (RT-qPCR) (Xiong et al., 2021), immunohistochemistry (IHC), and (Huang et al., 2019) histology. The objectives of this study were (Pittenger et al., 2019): to assess the osteochondrogenic induction potential of the muscle tissue after 1 month of continuous application of BMP-2 and/or TGF-β3 and/or Noggin and (Xiong et al., 2021) to investigate the so far unclear interaction mechanisms between the three growth factors during the endochondral bone induction process and if there are unique interactions in respect to tissue morphogenesis between the various growth factor combinations.

## 2 Results

### 2.1 Chondrogenesis

**FIGURE 1:**
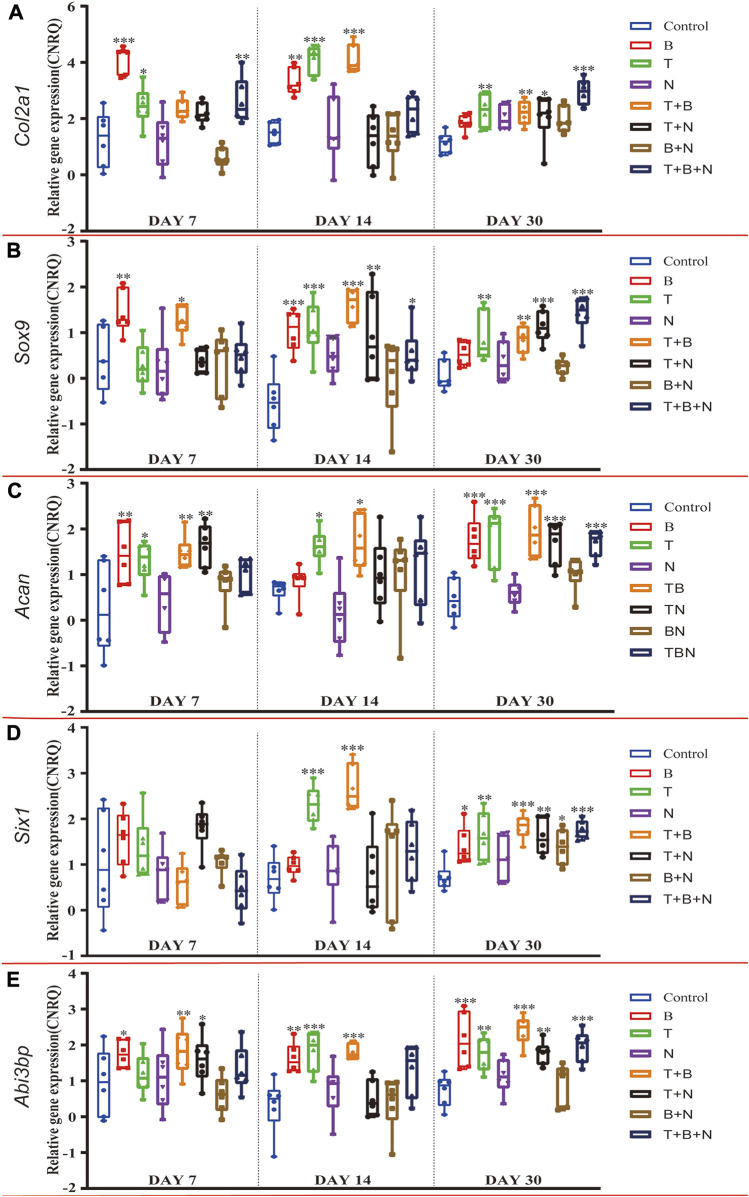
The relative gene expression of **(A)** *Col2a1*, **(B)** *Sox9*, **(C)** *Acan*, **(D)** *Six1* and **(E)** *Abi3bp* at 7, 14, and 30 days, which were shown as CNRQ. The asterisks indicate that the stimulated group is statistically significant compared to the control group. The baseline number 0 indicates non-cultured fresh tissue was used as the normalization parameter. (n = 6; *p < 0.05; **p < 0.01; ***p < 0.001).

**FIGURE 2:**
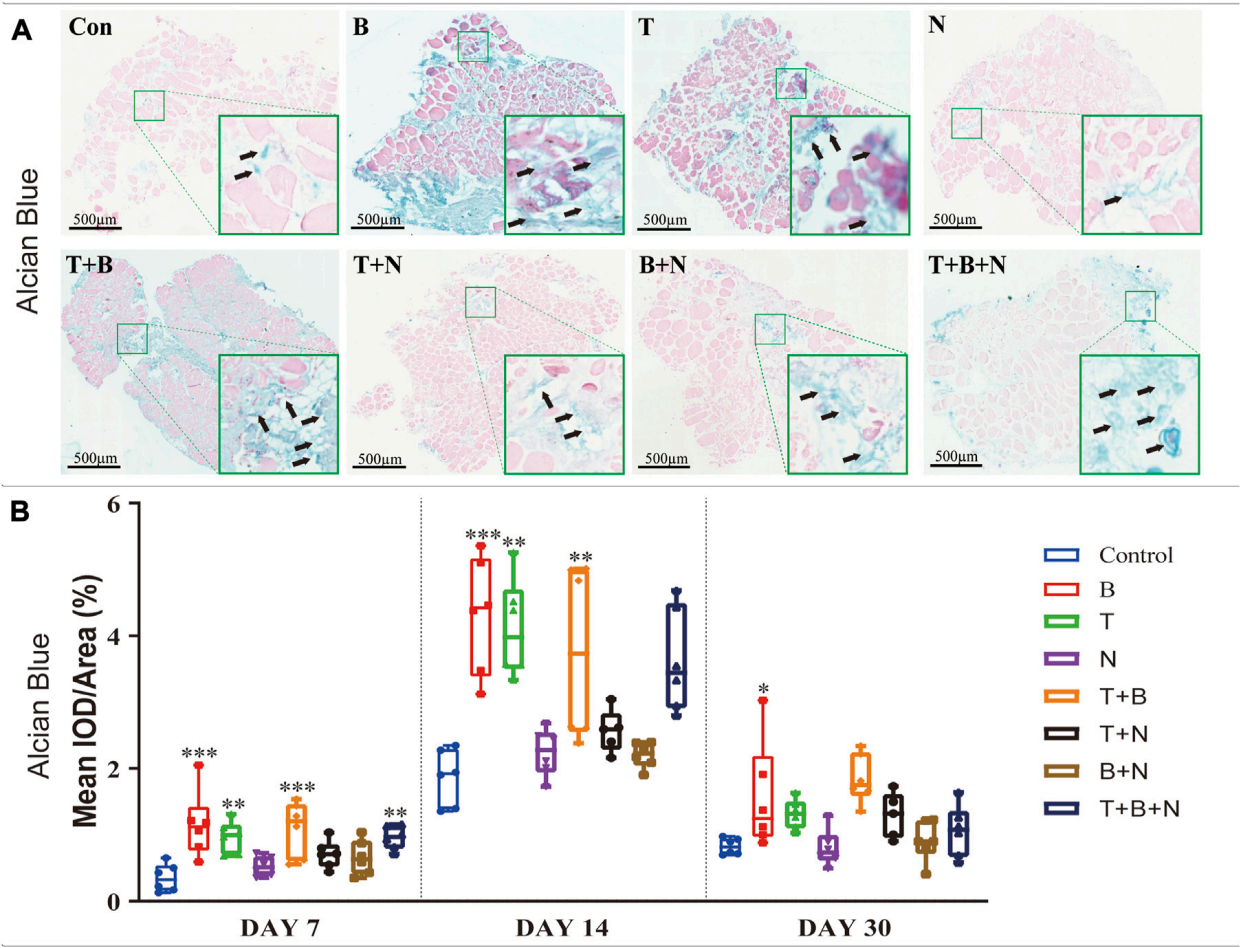
The staining results of Alcian Blue in each group. **(A)** Staining results on day 30; the positive staining color was blue (marked by black arrows). **(B)** Histomorphometrical assessment; the result was shown as Mean IOD/Area. Control group vs. stimulated groups at 7, 14, and 30 days; the asterisks indicate that the stimulated group is statistically significant compared to the control group. (Magnification: ×40; n = 6; *p < 0.05; **p < 0.01; ***p < 0.001).

**FIGURE 3:**
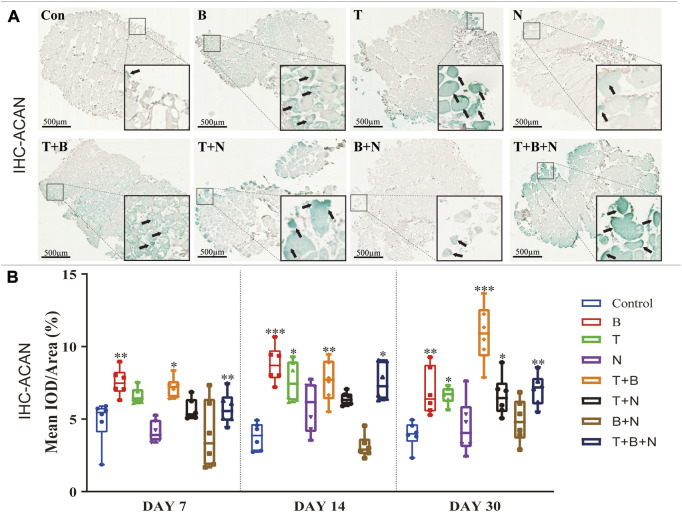
The staining results of ACAN antigen in IHC in each group. **(A)** Staining results on day 30; the positive staining color was green (marked by black arrows). **(B)** Histomorphometrical assessment; the result was shown as Mean IOD/Area. Control group vs. stimulated groups at 7, 14, and 30 days; the asterisks indicate that the stimulated group is statistically significant compared to the control group. (Magnification: ×40; n = 6; *p < 0.05; **p < 0.01; ***p < 0.001).

The chondrogenesis was evaluated at the following levels: gene expression (Figure 1), Alcian Blue (Figure 2) and IHC-ACAN staining (Figure 3, Table 1).

**TABLE 1:** The summarized results of/between these three growth factors.

Growth factor application	Effect/Result (culture period vs reaction intensity chondrogenesis)			Effect/Result (culture period vs reaction intensity for osteogenesis)			Interpretation
	Day 7	Day 14	Day 30	Day 7	Day 14	Day 30	
BMP-2	**+++**	**++**	**+**	**+++**	**++**	**++**	BMP-2 may function as an initiator only with a short effect period
TGF-β3	**+**	**+++**	**+++**	**+**	**+++**	**+++**	TGF-β3 affects tissue morphogenesis mid-late term
Noggin	**−**	**−**	**−**	**−**	**−**	**−**	Noggin inhibits tissue morphogenesis
TGF-β3+BMP-2	**−/+**	**+++**	**+++**	**−**	**++**	**++**	Early stage antagonism that inverts to synergism at later stages
TGF-β3+Noggin	**++**	**−**	**+++**	**+**	**−**	**++**	Synergistic stimulatory effect at early and late culturing stages with periods of inhibition in between (modulation of tissue morphogenesis?)
BMP-2+Noggin	**−**	**−**	**−**	**−**	**−**	**−**	Noggin inhibits BMP-2 function, prevents tissue differentiation
TGF-β3+BMP-2 +Noggin	**−**	**++**	**+++**	**−**	**−**	**+++**	Noggin synergizes with BMP-2 only at specific periods and when in the presence of TGF-β3

Remarks: little to no reaction; **−/+** *a* weakish reaction; **+** low reacting; **++** mid reaction; **+++** high reaction.

In order to evaluate chondrogenic gene expression in response to single or combined exposure of the modulating factors TGF-β3 (T), BMP-2 (B) and Noggin (N) in the muscle tissue model, temporal gene expression of cartilage-specific marker genes were analyzed by RT-qPCR. For the fibrillar collagen marker gene *Col2a1*, the control group showed similar, moderately upregulated expression on each day compared to the non-cultured, fresh muscle tissue (Figure 1A). The B group had the highest relative *Col2a1* expression on day 7, which was significantly upregulated compared to the control, similar to T and T + B + N groups. The combination of T + B increased *Col2a1* expression significantly only on days 14 and 30. The T + B and T + B + N groups showed the highest relative gene expression on days 14 and 30, respectively. Except for day 30 of the T + N and T + B + N groups and day 7 of the T + B + N group, *Col2a1* gene expression did not change significantly in all other N-treated groups compared to the control (Figure 1A, Supplementary Table S1).

The expression of the chondrogenic master transcription factor *Sox9* peaked in groups B, T + B, and T + B + N on days 7, 14, and 30, respectively, and all three groups showed significant differences compared to the control. The T group exhibited the highest *Sox9* expression on day 14 and showed significant upregulation on days 14 and 30. Among N-treated groups, significant *Sox9* upregulation was observed in the N group on day 14 and in the T + N and T + B + N groups on days 14 and 30, respectively (Figure 1B, Supplementary Table S1). For the major proteoglycan marker *Acan*, significantly upregulated gene expression was found in the B-, T-, T + B-, and T + N-stimulated groups on day 7 compared to the control. On day 14, only the T and T + B groups showed significant upregulation. On day 30, all groups except N and B + N presented significant *Acan* upregulation compared to the control (Figure 1C, Supplementary Table S1).

It has been previously shown that transcripts of *Six1* and *Abi3bp* are enriched in articular chondrocytes compared to growth plate chondrocytes; therefore, these genes have been proposed as markers for articular cartilage (Lee et al., 2021). In our muscle tissue model, we found that on day 30, *Six1* gene expression was upregulated in all treated groups compared to the control, except N (Figure 1, Supplementary Table S1), and *Abi3bp* was upregulated in all treated groups, except N and N + B (Figure 1, Supplementary Table S2). In the case of *Six1*, there was no significant difference in gene expression relative to control in either group on day 7, while on day 14, only the T and T + B groups displayed significant *Six1* upregulation (Figure 1D, Supplementary Table S1). Significantly upregulated expression of *Abi3bp* was found in the B, B + T, and T + N groups on day 7 and in the B, B + T, T + N, and T + B + N groups on day 14. Moreover, *Abi3bp* gene expression in the N and B + N groups showed no significant difference compared with control at all three time points (Figure 1E, Supplementary Table S2).

Alcian Blue staining was used to assess chondrogenesis in the cultured muscle tissue samples. Increased staining areas in blue were observed near the fascia or in the intercellular region of the muscle when stimulated by T, B, T + B, and T + B + N at all detection time points compared to the control (Figure 2A). Histomorphometric comparisons with the control showed that the B and T + B groups presented a significantly increased positive reaction area at all three-time points, while the T- and T + B + N-stimulated groups displayed significant positive reactions on days 7 and 14. On the other hand, all groups showed the strongest positive Alcian Blue staining results at 14 days, while the N and B + N groups consistently showed no significant differences compared with the control (Figure 2B, Supplementary Table S4).

IHC-ACAN staining was carried out to show the presence of ACAN antigens. A positive antigen–antibody interaction would be stained in a green color, which could be observed in close proximity to the fascia or in the intercellular region of the muscle when stimulated by B, T, T + B, T + N, and T + B + N at all detection time points (Figure 3A). The histomorphometrical assessments of IHC-ACAN staining showed that the B and T + B groups presented a positive reaction during all three-time points, while the T and T + B + N groups displayed a positive reaction on days 14 and 30. In addition, the T + N group also exhibited a significant difference on day 30. Additionally, no B + N group showed a significant difference (Figure 3B, Supplementary Table S4).

### 2.2 Osteogenesis

**FIGURE 4:**
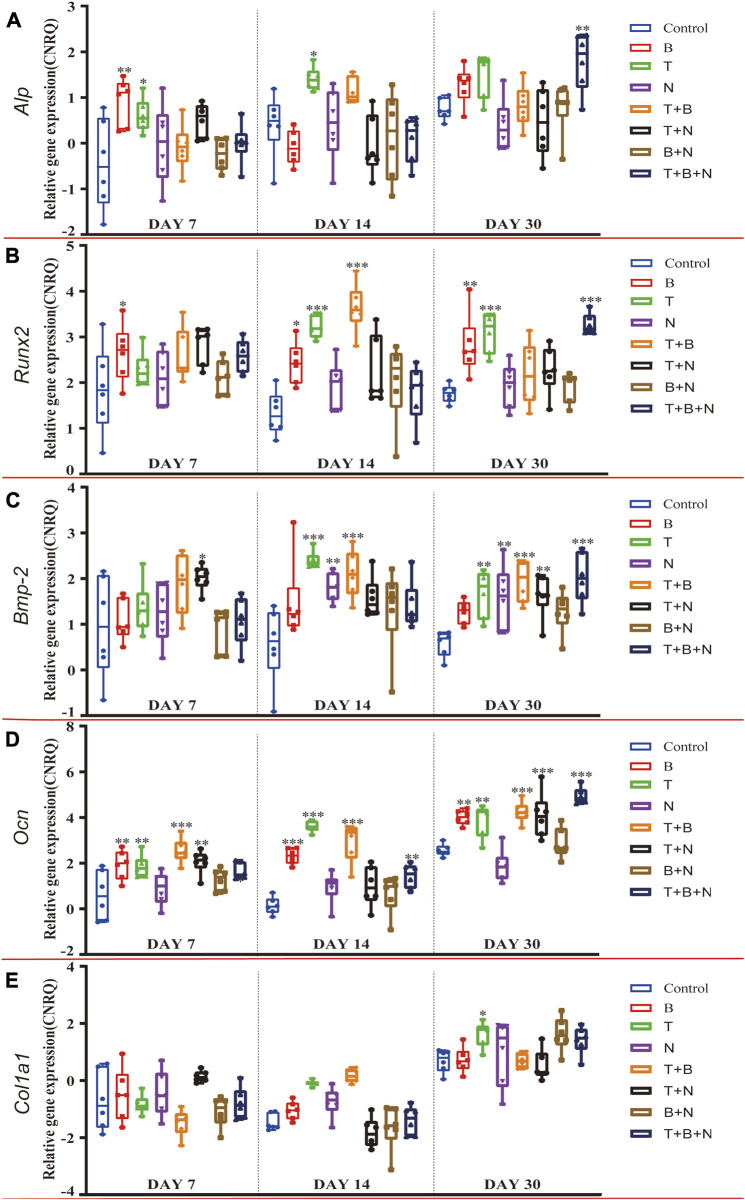
The relative gene expression of **(A)** *Alp,* **(B)** *Runx2*, **(C)** *Bmp-2*, **(D)** *Ocn*, and **(E)** *Col1a1* at 7, 14, and 30 days, which were shown as CNRQ. The asterisks indicate that the stimulated group is statistically significant compared to the control group. The baseline number 0 indicates non-cultured fresh tissue was used as the normalization parameter. (n = 6; *p < 0.05; **p < 0.01; ***p < 0.001).

**FIGURE 5:**
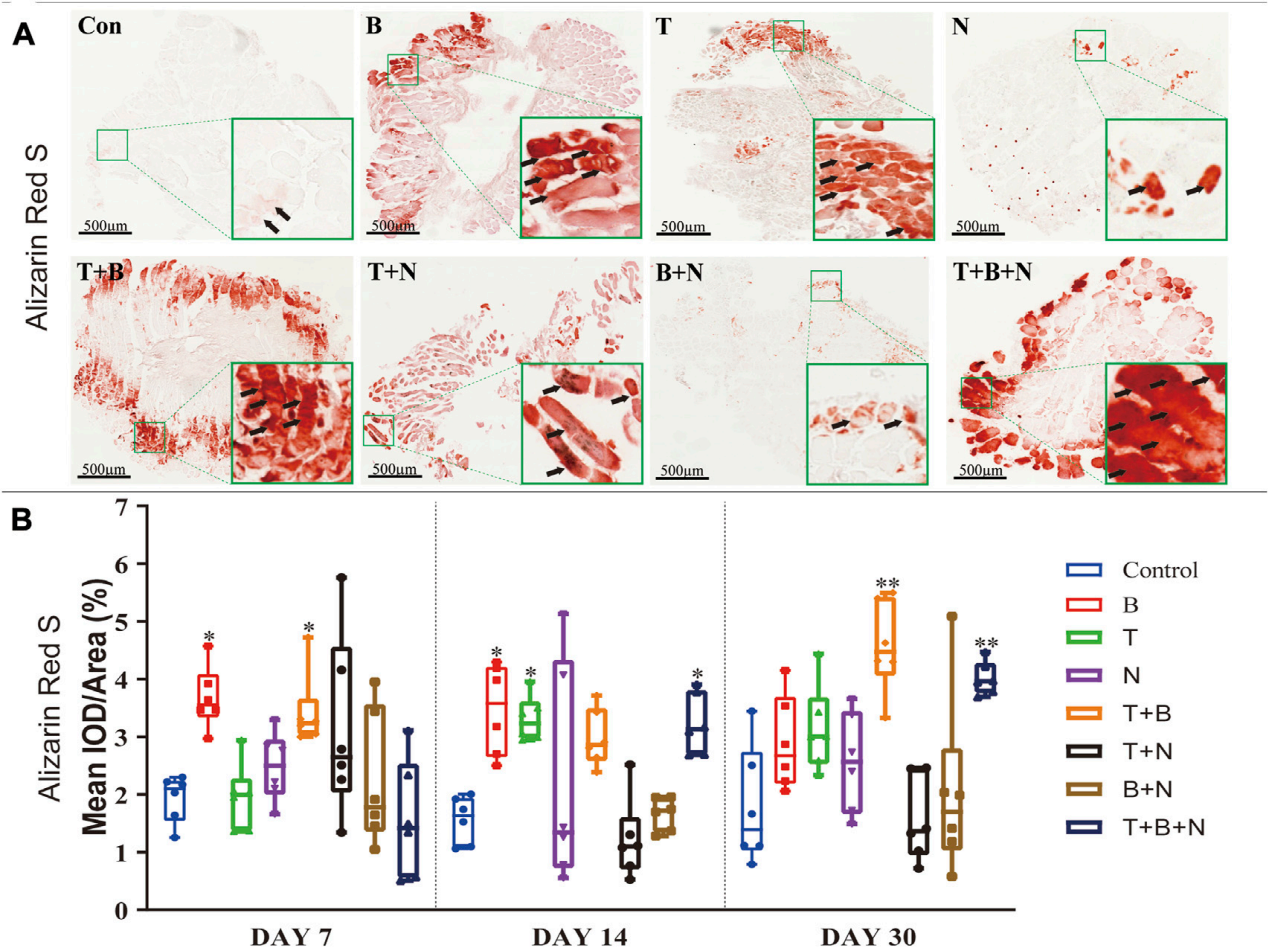
The staining results of Alizarin Red S in each group. **(A)** Staining results on day 30; the positive staining color was red (marked by black arrows). **(B)** Histomorphometrical assessment; the result was shown as Mean IOD/Area. Control group vs. stimulated groups at 7, 14, and 30 days; the asterisks indicate that the stimulated group is statistically significant compared to the control group. (Magnification: ×40; n = 6; *p < 0.05; **p < 0.01; ***p < 0.001).

**FIGURE 6:**
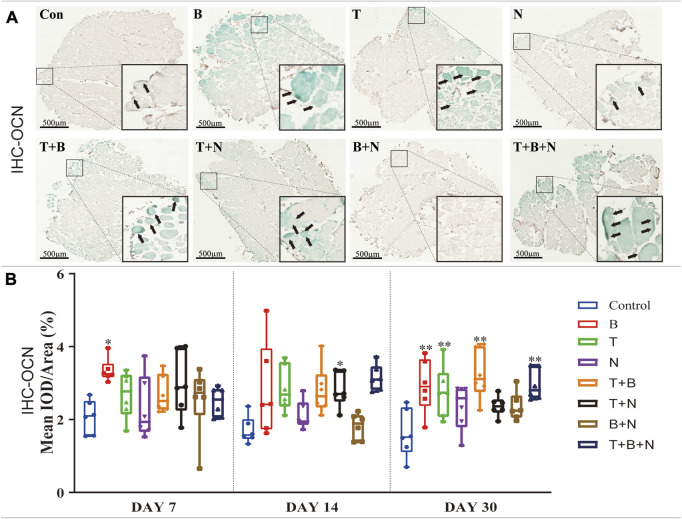
The staining results of the OCN antigen in the IHC in each group. **(A)** Staining results on day 30; the positive staining color was green (marked by black arrows). **(B)** Histomorphometrical assessment; the result was shown as Mean IOD/Area. Control group vs. stimulated groups at 7, 14, and 30 days; the asterisks indicate that the stimulated group is statistically significant compared to the control group. (Magnification: ×40; n = 6; *p < 0.05; **p < 0.01; ***p < 0.001).

The osteogenesis was evaluated at the following levels: gene expression (Figure 4), Alizarin Red S (Figure 5) and IHC-OCN staining (Figure 6).

For *Alp*, the B group showed the highest relative gene expression on day 7, which was significantly upregulated, along with the T and T + B + N groups. Additionally, the T-induced group was the only one that presented a significant *Alp* expression on day 14, and only the T + N and T + B + N groups demonstrated a significant upregulation of *Alp* expression. On the other hand, *Alp* expression in all N-involved groups showed no significant difference (Figure 4A, Supplementary Table S2). For the relative *Runx2* gene expression, the T + N group became the only group that showed a significant difference at 7 days, while at 14 days, the significantly upregulated *Runx2* gene expression was found in B, T, and T + B groups. By 30 days, the B, T, and T + B + N groups showed high and significant gene expression. In addition, except for the 7-day T + N and 30-day T + B + N groups, *Runx2* gene expression in all other Noggin-involved groups was not significant (Figure 4B, Supplementary Table S2). For the relative *Bmp-2* gene expression, the T + N group showed a significant difference across all three-time points; in addition, T, N, and T + B groups presented significantly upregulated *Bmp-2* gene expression at both day 14 and 30. Moreover, the T + B + N group showed the highest and most significant gene expression on day 30. In addition, all B + N groups showed non-significant *Bmp-2* gene expression (Figure 4C; Supplementary Table S3). For the relative *Ocn* gene expression, the B, T, T + B, and T + B + N groups all presented significant upregulation among the three-time points. Additionally, the T + N group also showed significant *Ocn* gene expression on days 7 and 30, but a non-significant difference was found on day 14. Furthermore, the N and B + N groups exhibited non-significant *Ocn* gene expression all the time (Figure 4D, Supplementary Table S3). For *Col1a1*, no treatment group showed upregulated relative gene expression at 7 days, while the T and T + B groups were significantly upregulated at 14 days. By 30 days, although most stimulated groups showed upregulation of *Col1a1* gene expression, only the T group exhibited a significant difference (Figure 4E, Supplementary Table S3).

Alizarin Red S staining was applied to show the depositions of calcium ions in tissues as a measure of osteogenesis. Under B, T, T + B, T + N, and T + B + N stimulation, areas of positive staining in red were observed in close proximity to the fascia or intercellular regions of the muscle at all detection time points (Figure 5A). Histomorphometric evaluation of Alizarin Red S staining showed that the B group presented a significant positive reaction on days 7 and 14, while the T group only displayed a significant positive reaction on day 14 compared to the control. In addition, the T + B group displayed a positive reaction on days 7 and 30. Moreover, the T + B + N group showed significant stimulation of osteogenesis from day 14 until day 30. The N and B + N groups consistently showed no significant difference compared to the control (Figure 5B, Supplementary Table S4).

IHC-OCN staining was carried out to show the presence of the OCN antigen. Under the stimulation of B, T, T + B, T + N, and T + B + N, areas of positive staining were observed in close proximity to the fascia or intercellular regions of the muscle with green color at all detection time points (Figure 6A). The histomorphometrical assessment of IHC-OCN staining showed that, although there was a generally high positive reaction on day 7, the B group was the only one that had a significant difference. In addition, the T + B + N group became the only significant positive stimulation group at 14 days, while the B, T, T + B, and T + B + N groups all showed significant differences by day 30. Additionally, no B + N group showed a significant difference (Figure 6B, Supplementary Table S4).

### 2.3 Heat map analysis

**FIGURE 7:**
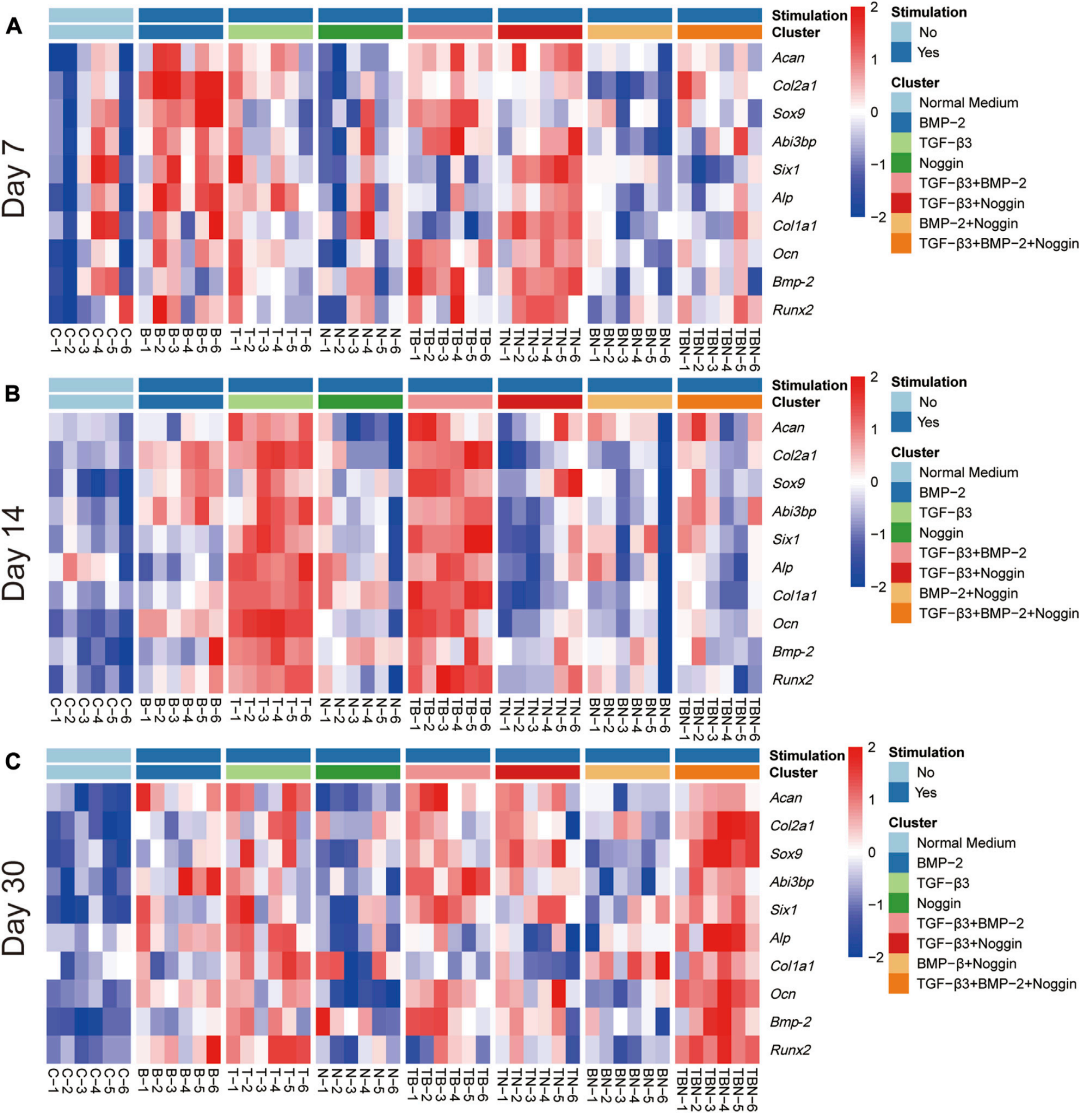
Heat map of gene expression. All relative gene expression at 7 **(A)**, 14 **(B)** and 30 days **(C)**. *Acan = Aggrecan, Col2a1= Collagen type II alpha 1, Sox9 = Sex determining region Y (SRY)-box transcription factor 9, Six1= Six homeobox 1, Abi3bp = Abi family member 3 binding protein, Runx2 = Runx family transcription factor 2, Alp= Alkaline phosphatase, Bmp-2 = Bone morphogenetic protein-2, Ocn = Osteocalcin, Col1a1 = Collagen type I alpha 1 chain*; (n = 6).

**FIGURE 8:**
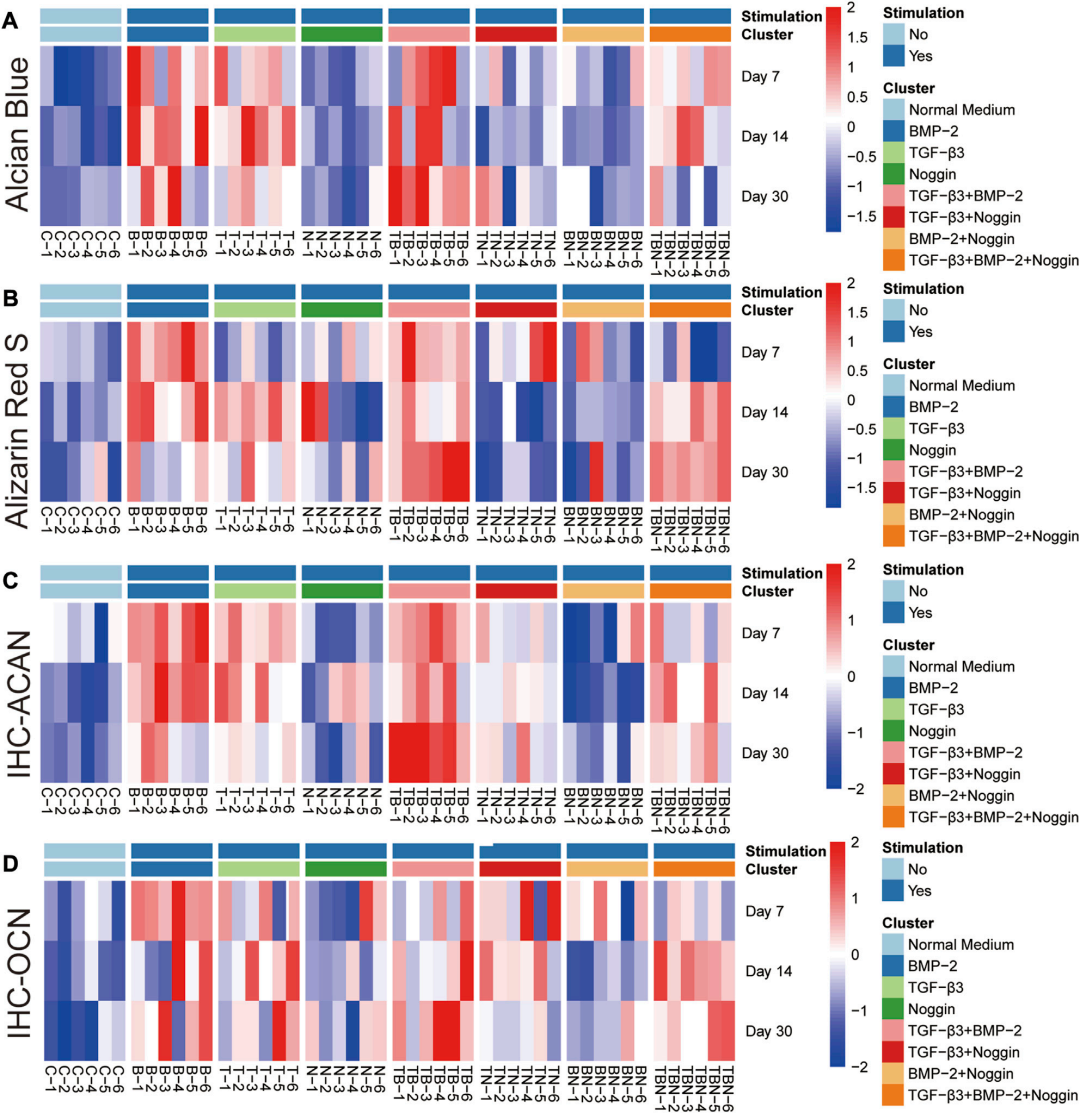
Heat map of histomorphometrical analyses. **(A)** Alcian Blue staining. **(B)** Alizarin Red S staining. **(C)** IHC-ACAN staining. **(D)** IHC-OCN staining. IHC = Immunohistochemistry, ACAN = Aggrecan, OCN = Osteocalcin; (n = 6).

The heat map analysis of gene expression and histomorphometrical data are represented in Figure 7 and Figure 8, respectively. The heat map is a summary of the results (Table 1) indicating where significant differences exist in the gene expression and tissue development.

The heat map of gene expression showed that the B- and T + N-stimulated groups promoted relatively high gene expression at 7 days (Figure 7A); the T- and T + B-stimulated groups displayed relatively high gene expression at 14 days (Figure 7B); while the stimulation of T, T + B, T + N, and T + B + N exhibited high gene expression at 30 days (Figure 7C). Compared to 7 and 30 days, stimulation by T + N resulted in less gene expression at 14 days. Additionally, the N and B + N groups did not show high gene expression at all time periods (Figure 7).

As seen in all the histomorphometrical analyses of the heat map, the B and T + B groups presented the most robust positive response results compared to the other participating groups. However, the single B group performed better in the early phase (7 and 14 days), while the combined T + B group was more dominant in the late phase (30 days). In addition, stimulation by T alone also displayed positive results, although slightly weaker than the T + B combination. Furthermore, the T + B + N group resulted in relatively higher positive reactions in all staining at late stages (at 14 and 30 days), except for the 30-day Alcian Blue staining (Figure 8). All results were summarized in Table 1.

## 3 Discussion

The TGF-β/BMP signaling pathway is an important thread essential for osteochondrogenic tissue formation (Zhou et al., 2005; Bami et al., 2016; Izadpanahi et al., 2018). Endochondral bone development and articular chondrogenesis are closely regulated by diverse growth factors (Chung et al., 2001; Liao et al., 2014). Generally, the results of this study showed that both chondrogenic and osteogenic-related genes underwent significant changes over the 30 days of *in vitro* culturing with TGF-β3 and/or BMP-2 groups and the TGF-β3+BMP-2+Noggin sets. Combined with the histomorphometrical results, the findings suggest that our muscle tissue may be undergoing an osteochondrogenic process, favoring an articular to endochondral bone transdifferentiation activity. The positive IHC-ACAN and the strong Alcian Blue staining in conjunction with the significant upregulation of *Sox9, Acan,* and *Col2α1* genes, in addition to the upregulation of articular cartilage genes *Abi3bp* and *Six1*, suggest that a form of articular chondrogenesis was being induced (Xiong et al., 2020). Whilst it remains unclear if this is proper articular cartilage or a specialized undiscovered form of the process, its detection corroborates the principle that the process of endochondral bone formation is always accompanied by the appearance of hyaline cartilage (Blumer et al., 2005; Grässel and Aszódi, 2016). On the other hand, the positive results of IHC-OCN and Alizarin Red S staining showed the abundant presence of OCN and calcium deposition, inferring that a bone-related ECM was either also being formed or a transition was underway from the chondrogenic tissue to that of a bone-like tissue (McLeod, 1980; Ding et al., 2019). We believed this to be the case, as the increases in *Runx2, Alp, Ocn, Bmp-2,* and *Col1α1* gene expressions over the culturing periods were indicative of a trend towards osteogenic morphogenesis (Karsenty et al., 2009; Scott et al., 2012).

Though this seemed to be a general trend among the various growth factor groups analyzed, marked differences were also recorded. The present research experiment verified that both TGF-β3 and BMP-2 alone could initiate osteochondrogenesis. Especially *Sox9* and *Runx2,* master transcription factors for chondrogenesis and osteogenesis, respectively (Eames et al., 2004; Zhang et al., 2013), showed overlapping and significantly increased expressional regulation. On day 7, *Sox9* was positively expressed in the single BMP-2 group, while no significant result was detected for *Runx2* gene expression. This result was consistent with many previous studies that *Sox9* and *Runx2* play a reciprocal inhibitory role during osteo-chondrogenesis to influence mesenchymal cell fate (Yamashita et al., 2009; Cheng and Genever, 2010). During the early chondrogenic differentiation stage, BMP-2-induced *Runx2* expression was suppressed by *Sox9* to inhibit the subsequent endochondral ossification process and maintain the hyaline cartilage phenotype (Zhou et al., 2006; Liao et al., 2014). However, *Sox9* also contributed to BMP-2-induced osteogenic differentiation since *Sox9* silencing causes reduced osteogenesis in bone-marrow-derived mesenchymal stem cells (BMSCs) (Zhao, 2008; Fang et al., 2019). Alternatively, the groups treated with TGF-β3 only showed the positive upregulation of *Bmp-2* and *Runx2* gene expressions on days 14 and 30, confirming previous claims by Klar et al. (2014) and other studies that TGF-β3 seems to be able to regulate osteogenesis by modulating endogenous *Bmp-2* levels, followed by increased *Runx2* expression (Wang et al., 2016).

For the TGF-β3+BMP-2 groups, both synergistic and antagonistic activities were discovered that occurred at specific temporal culturing stages of our *in vitro* model. From day 0 to day 7 and 14, the addition of TGF-β3 blocked most of the BMP-2 gene and protein upregulation that normally would occur if TGF-β3 were absent. In relation to the inhibitory effects, it is known that both TGF-β3 and BMP-2 have similar receptor binding mechanisms, inferring that competitive inhibition of the TGF-βs and BMPs receptors is possible (Keller et al., 2011). Alternatively, TGF-βs could be blocking BMP signaling transduction by forming mix-linked Smad1/5-Smad3 inhibitory complexes (Daly et al., 2008; van der Kraan et al., 2012), or it could be that TGF-β3-induced inhibitory Smad6 or Smad7 are also interfering with the BMP signaling pathway (Keller et al., 2011). This has been well-described by various scientists. For instance, Ehnert et al. (2010); Ehnert et al. (2012) showed that Smad1/5/8-mediated BMP-2 and -7 signaling could be blocked entirely by adding recombinant human TGF-β in primary human osteoblasts. Similarly, Mehlhorn et al. (2007) presented that BMP-2 induced chondrogenesis and osteogenesis in adipocyte-derived stem cells could be prevented by simultaneously applying any of the three TGF-β isoforms.

However, the synergistic activities between TGF-β and BMP signaling were also found in the same tissue model system, but only during the later 30-day stages of culture. From 14 to 30 days, most detected genes and proteins were significantly higher upregulated in the TGF-β3+BMP-2 group than either the TGF-β3 or BMP-2 groups (Figure 7B). The possible underlying mechanisms of the synergistic effect, and those at specific time points, could be that TGF-βs switch function over time. This would suggest that TGF-β3 can alternate between being a competitive inhibitor of the BMPs pathways to being an activator of cellular stimulation, at specific time points, either due to changes in concentration or intrinsic cellular alteration. Apart from binding ALK5 to stimulate the canonical Smad2/3 signaling pathway, TGF-βs can also exert functions *via* activating the BMP signaling pathway by associating with ALK1 and ALK2 directly and then triggering Smad1/5/8 for signal transmission (Wrighton et al., 2009; Keller et al., 2011). The synergistic effect between TGF-βs and BMPs is well known (Wu et al., 2016). However, if growth factors change function with time, switching roles based on cellular activity or differentiation/transformation changes, this needs to be further analyzed in future studies. This is especially critical given that our Noggin results showed a similar function switching from inhibitor to stimulator.

The ectopic application of Noggin in our experiment confirms that one of the roles of Noggin is to antagonize BMP-2-induced osteochondrogenic differentiation. Nearly all applications of Noggin alone and BMP-2+Noggin combined presented insignificant expressional changes, both at the gene and protein levels and at all culturing time points. As a key natural BMPs antagonist, Noggin can specifically bind BMP-2, -4, -5, -6, and -7 with several degrees of affinity, including GDF-5/-6, yet provides little to no binding affinity to the other TGF family members (Smith and Harland, 1992; Song et al., 2010). However, our experiment counteracts this assumption as Noggin seemed to actively inhibit exogenously applied TGF-β3 growth factor functioning, since Noggin prevented the upregulation of all genes that TGF-β3 normally activated on day 14 (Figure 7B). Indeed, the inhibitory effect of Noggin on TGF-β3 has been discovered and reported by many scholars. Nakayama et al. (2003) showed that Noggin could block TGF-β3 induced chondrogenesis, suggesting a BMP-associated pathway was involved. In addition, Bayramov et al. (2011) put forward a novel inhibitory function of Noggin by demonstrating that, in addition to BMPs, several non-BMP ligands, such as Activin B, Xnr2, and Xnr4, can also be antagonized by Noggin, albeit less efficiently. Interestingly, these blocked non-BMP ligands regulate specific genes’ transcription through cytoplasmic Smad2/3. This point may indicate another link between TGF-β3 and Noggin regarding non-BMP ligands and downstream effectors Smad2/3. From this and in conjunction with our results, we deduce that the application of Noggin can, at specific time points, inhibit the differentiation function of both BMP-2 and TGF-β3 signaling.

However, the inhibition effect of Noggin + TGF-β3 was not observed at day 7 nor day 30. Instead, at these time points, our results showed that most of the gene and protein expression markers increased significantly, promoting the idea that signals, whether they be growth factors or antagonists such as Noggin, possess various roles that are not limited to a single function but are temporally dependent. Indeed, our results suggest a positive function of Noggin in osteo-chondrogenesis at specific temporal stages. Interestingly, a similarly positive result could also be observed with our TGF-β3+BMP-2+Noggin groups at day 30 (Figures 7A, C; Figures 8B–D). While this interpretation does go against the traditional concept that Noggin should inhibit osteo-chondrogenesis, Noggin’s positive stimulatory functions have been reported. For instance, Chen et al. (2012) proposed that Noggin can stimulate human MSCs osteogenesis, as the suppression of significantly reduced BMP-2-induced ALP activity. Rifas (2007) made a similar observation showing that Noggin could induce ALP action and upregulate *Bmp-2* and *Ocn* gene expression. Unusually, other than these ordinary osteogenic markers, they also found increased ActRII expression (Rifas, 2007). Furthermore, Hashimi (2019) found that exogenous Noggin treatment could induce osteogenesis by binding to and stimulating the BMP-2 receptor (Hashimi, 2019). Taken together with our discoveries, this would suggest that Noggin may perform a stimulatory role during specific temporal stages of osteo-chondrogenesis development, especially when it is in the presence of TGF-β3. Future research needs to investigate this more clearly, as there is a definitive lack of knowledge regarding the temporal behavior of growth factors and inhibitors over time and at which time points signals change their function.

Over the course of nearly 3 decades, research into the possible mechanisms for the formation and regenerating of bone or articular cartilage tissue, have yielded few clinically relevant solutions (Wei and Dai, 2021). Whilst a large spectrum of regenerative scientists and tissue engineers are trying to find new alternatives, Klar (2018) possibly provided one of the most prudent solutions to solving this dilemma, being that “all of the relevant signals and their interactions had not been fully established”. This inferred that gaps in the knowledge exist in how ligands are activated and how their effect changes over time when affecting tissue development. Indeed, the current work not only establishes that our knowledge on signals and their behavior over the course of time changes drastically between stimulation, antagonism, and regulation, but that with the correct combination of signals any tissue could be indirectly (*in vitro*) or directly (*in vivo*) transformed into whatever tissue/organ we desire. The clinical implications of such information would be invaluable for future therapies as whole organs or even limbs could be fully grown from excess damaged tissue areas or excess tissue types be biological recycled to form new tissues/organs (Betz et al., 2009; Xiong et al., 2020).

Whilst our results did show some critical new discoveries and possible avenues of research, the study also had certain limitations. A critical limitation was that we did not consider the effect of the dose gradient of the applied growth factors on the experimental results. Whilst we tried to choose a dose that would elicit a response without causing inhibition, some studies have reported that the TGF-β superfamily factors serve as a double-edged sword in DNA synthesis (Chen et al., 1991; Harris et al., 1994). For example, a low concentration of TGF-β can promote osteogenic differentiation but inhibit it at a high concentration (Karst et al., 2004; Crane et al., 2016). In addition, low doses of active TGF-β have also been shown in chondrocytes to preferentially signal through the Smad2/3 pathway, while the Smad1/5 pathway becomes predominant at high doses (Finnson et al., 2008; Blaney Davidson et al., 2009)**.** In addition, that BMP-2 controls bone formation in a concentration-dependent manner has also been demonstrated in bone TE studies (Meinel et al., 2006; Shi et al., 2012; Dang et al., 2016). Thus, an appropriate molecular concentration may play a vital role in a differentiation system as time progresses. Subsequently, another limitation was that we did not apply the growth factors in a truly temporal manner, i.e., first BMP-2 for 2 days then TGF-β3 for 2 days, etc., nor adjust the application order. Iwakura et al. (2013) established that morphogen treatment order could produce varying effects. Applying growth factors, such as BMP-7 followed by TGF-β1, resulted in more effective chondrogenesis than TGF-β1 following BMP-7. On the other hand, although numerous types of cells give the muscle tissue the possibility of multiple differentiation, it also increases the uncertainty of its differentiation direction. It is challenging to match various differentiated phenotypes with corresponding cell types in such a complex 3D cellular assembly. As such, a comparison between the muscle tissue explant and a specific single cell type, such as satellite cells or myoblasts, may be necessary to be conducted, especially in a 3D pellet culture condition, to confirm the superiority of this muscle tissue induction model. Moreover, the increasing trend of gene expression in the control group, although not significantly different compared to the 0-day sample (baseline), might suggest that the induced phenotypes were not absolutely derived from the exogenous molecules. One of the explanations may be that the FBS in the normal medium provided some supplementary signals for differentiation. The other reason may be attributed to the injury from tissue excision since the trauma-induced various BMPs expression and followed heterotopic ossification have been verified by many investigators (Li, 2020; Strong et al., 2021). Finally, to achieve a more realistic *in vitro* physiological simulation system, mechanical and even electrochemical stimulation, as directed by nerves, should also be considered as a complement to biochemical cues in this muscle-tissue-based model because they can also play essential and unique roles as temporal biophysical signals to participate in cellular activities (Boonen et al., 2010; Maleiner et al., 2018; Urdeitx and Doweidar, 2020).

## 4 Materials and methods

### 4.1 Collection of muscle tissue samples

The rectus abdominis muscle tissue was collected from two Fischer-344 adult *Rattus norvegicus* (Charles River Wiga, Sulzbach, Germany). The animals were sacrificed with an excess of isoflurane (Abbot, Chicago, United States) and disinfected with 10% povidone-iodine (Betadine, Bonn, Germany) and 75% alcohol (Apotheke Großhadern, Munich, Germany). Under a sterile environment, the harvested muscle was incubated in graded concentrations of penicillin and streptomycin (2% and 1%) (A2213; P/S, Biochrom GmbH, Berlin, Germany) in Alpha-Medium (Biochrom GmbH, Berlin, Germany) for 20min, respectively. Then, 288 fragments of the tissue 4 mm in diameter were obtained with a specific biopsy punch (PFM medical, Cologne, Germany). The rules and regulations of the Animal Protection Laboratory Animal Regulations (2013) of the European Directive 2010/63/EU Act were strictly complied with during the above procedures. The experiments were also approved by the Animal ethics research committee of the Ludwig Maximillian University of Munich (LMU), Bavaria, Germany Tierschutzgesetz §1/§4/§17 (https://www.gesetze-im-internet.de/tierschg/TierSchG.pdf) with regard to animal usage for pure tissue or organ harvesting only.

### 4.2 Muscle tissue culture

The muscle tissue biopsies (n = 288) were cultured in 96-well plates (Thermo Fisher Scientific, Waltham, MA, United States) in normal culture medium (containing Alpha-Medium, 1% P/S, 0.02 mM/mL L-glutamine (Biochrom GmbH, Berlin, Germany) and 15% fetal bovine serum (FBS; Biochrom GmbH, Berlin, Germany)) for 48 h in a humidified incubator with 5% CO_2_ at 37°C to allow for the cells in the tissue to recover. The muscle tissue fragments were then divided into eight independent treatment groups:

(Pittenger et al., 2019) Control **(Con)** group, containing the normal culture medium (Xiong et al., 2021); Rat BMP-2 **(B)** group, containing the normal culture medium and 50 ng/mL BMP-2 (CUSABIO, United States) (Huang et al., 2019); Rat TGF-β3 **(T)** group, containing the normal culture medium and 50 ng/mL TGF-β3 (Cloud-Clone Corp, United States) (Huynh et al., 2019); Rat Noggin **(N)** group, containing the normal culture medium and 50 ng/mL Noggin (Cloud-Clone Corp, United States) (Sheehy et al., 2013); TGF-β3+BMP-2 **(T + B)** group, containing the normal culture medium and 50 ng/mL TGFb3+50 ng/mL BMP-2 (Schaefer et al., 2002); TGF-β3+Noggin **(T + N)** group, containing the normal culture medium and 50 ng/mL TGF-β3+50 ng/mL Noggin (Alhadlaq and Mao, 2005); BMP-2+Noggin **(B + N)** group, containing the normal culture medium and 50 ng/mL BMP-2+50 ng/mL Noggin (Zhang et al., 2019); TGF-β3+BMP-2+Noggin **(T + B + N)** group, containing the normal culture medium and 50 ng/mL TGF-β3+50 ng/mL BMP-2+50 ng/mL Noggin.

Each modality had 36 samples that were divided up into quantitative genes (n = 6) as well as histological (n = 6) assessment groups and further into subsequent culture period lengths of 7, 14, and 30 days. In the end, for each treatment modality, there were always 6 muscle fragments for a given culture length and assessment method.

### 4.3 RT–qPCR

The minimum information for publication of quantitative real-time PCR experiments (MIQE) principles was strictly applied to guide the entire RT–qPCR procedure (Bustin and Wittwer, 2017). After flash freezing in liquid nitrogen, the harvested muscle tissue samples were homogenized by a mortar and pestle under an RNase-free work hood. The RNeasy^®^ Fibrous Tissue Mini Kit (Qiagen, Hilden, Germany) was used to extract and purify the total RNA. The obtained RNA samples had an A260/A280 ratio of 1.86–2.07 and a concentration of 76.7–123.7 ng/μL, which were measured by a NanoDrop^TM^Lite (Thermo Fisher Scientific, Waltham, MA, United States). Finally, a QuantiTect complementary DNA (cDNA) Synthesis Kit (Qiagen, Hilden, Germany) was applied according to their specialized protocol to conduct reverse transcription. The resulting cDNA was deposited at −20°C for subsequent qPCR analysis.

The qPCR process was performed on a LightCycler^®^ 96 Instrument (Roche, Switzerland), utilizing the FastStart Essential DNA Green Master and SYBR Green I Kit (Roche, Switzerland). The thermal cycling parameters were set in 3 min initial denaturation steps at 95°C; 40 cycles, including a denaturation step at 95°C for 10 s, an annealing step at 60°C for 15 s, and an extension step at 72°C for 30 s, respectively; and a final extension at 72°C for 5 min. The final reaction volume was 10 μL, consisting 2 μL cDNA (5 ng/μL), 1.8 μL RNase-free water, 5 μL Green Master, 0.6 μL forward primer, and 0.6 μL reverse primer. The primers of eight reference genes and ten target genes (Table 2) were designed and analyzed on the IDT website (https://eu.idtdna.com/site).

**TABLE 2:** The target and reference genes information.

	Gene name	Accession nr	Fwd. (5′-3′)	Rev. (5′-3′)
	*Actb*	NM_031144.3	AGCTATGAGCTGCCTGA	GGC​AGT​AAT​CTC​CTT​CTG​C
	*Rplp0*	BC001834.2	CAACCCAGCTCTGGAGA	CAGCTGGCACCTTATTGG
Reference genes	*Gapdh*	BC083511.1	CATGGGTGTGAACCATGA	TGTCATGGATGACCTTGG
	*Polr2e*	BC158787.1	GAC​CAT​CAA​GGT​GTA​CTG​C	CAG​CTC​CTG​CTG​TAG​AAA​C
	*Sdha*	NM_130428.1	GCG​GTA​TGA​GAC​CAG​TTA​TT	CCTGGCAAGGTAAACCAG
				
	*Acan*	NM_022190.1	CAAGTGGAGCCGTGTTT	TTT​AGG​TCT​TGG​AAG​CGA​G
	*Col2a1*	NM_012929.1	ATCCAGGGCTCCAATGA	TCTTCTGGAGTGCGGAA
	*Sox9*	NM_080403.1	CCA​GAG​AAC​GCA​CAT​CAA​G	ATA​CTG​ATG​TGG​CTG​GTG​G
	*Six1*	NM_053759.1	CAGGTTCTTGTGGTCGTT	TTTGGGATGGTTGTGAGG
Target genes	*Abi3bp*	XM_017598145.1	ACG​GGA​CAT​TCC​TCT​CAT​A	GGTGCCTGAGTTGTCTTT
	*Runx2*	NM_001278484.2	CCCAAGTGGCCACTTAC	CTGAGGCGGTCAGAGA
	*Alp*	NM_013059.2	CGACAGCAAGCCCAAG	AGACGCCCATACCATCT
	*Bmp-2*	NM_017178.1	GGAAGTGGCCCACTTAGA	TCA​CTA​GCA​GTG​GTC​TTA​CC
	*Ocn*	NM_013414.2	GCGACTCTGAGTCTGACA	GGCAACACATGCCCTAAA
	*Col1a1*	NM_053304.1	GGT​GAC​AGA​GGC​ATA​AAG​G	AGACCGTTGAGTCCATCT

*Actb = Actin beta, Rplp0 = Ribosomal protein lateral stalk subunit p0, Gapdh = Glyceraldehyde-3-phosphate dehydrogenase, Polr2e = RNA, polymerase II, subunit e, Sdha = Succinate dehydrogenase complex flavoprotein subunit a; Acan = Aggrecan, Col2a1= Collagen type II, alpha 1, Sox9 = Sex determining region Y (SRY)-box transcription factor 9, Six1= Six homeobox 1, Abi3bp = Abi family member 3 binding protein, Runx2 = Runx family transcription factor 2, Alp= Alkaline phosphatase, Bmp-2, Bone morphogenetic protein-2, Ocn = Osteocalcin, Col1a1 = Collagen type I alpha 1 chain*.

The GeNorm (http://medgen.ugent.be/wjvdesomp/genorm/) was applied to assess and select Glyceraldehyde-3-phosphate dehydrogenase (*Gapdh*); Succinate dehydrogenase complex flavoprotein subunit A (*Sdha*); Ribosomal protein lateral stalk subunit P0 (*Rplp0*); RNA polymerase II, I and III subunit E (*Polr2e*); and Actin beta (*Actb*) as the final reference genes (Table 1) for the subsequent gene expression calibration process. Targets included the chondrogenesis-associated genes collagen type II (*Col2a1*), SRY-box transcription factor 9 (*Sox9*), aggrecan (*Acan*), SIX homeobox 1 (*Six1*) and ABI family member 3 binding protein (*Abi3bp*), and the osteogenesis-associated genes Alkaline phosphatase (*Alp*), RUNX family transcription factor 2 (*Runx2*), Bone morphogenetic protein 2 (*Bmp-2*), osteocalcin (Ocn) and Collagen type I alpha 1 chain (*Col1a1*). The relative gene expression levels were characterized in calibrated normalized relative quantities (CNRQs), which were obtained by normalization with the pre-determined reference genes in the qBase + software (https://www.qbaseplus.com/), including the relevant endogenous control (fresh muscle tissue 0-day).

### 4.4 Histological and immunohistochemical (IHC) staining

Harvested samples for histological analysis were first placed in 4% paraformaldehyde (Microcos GmbH, Garching, Germany) for overnight fixation, followed by dehydration in Spin Tissue Processor-120 (Especialidades Médicas Myr, S.L., Tarragona, Spain), then embedded in paraffin blocks. Afterwards, 3 μm-thick sections were cut for subsequent staining.

Alcian Blue staining was used to evaluate the effectiveness of chondrogenesis in this study. Deparaffinized and hydrated sections were stained in 1% Alcian Blue solution (pH 2.5, Morphisto, Frankfurt, Germany) and counterstained in 0.1% Nuclear Fast Red solution (Morphisto, Frankfurt, Germany) and were then dehydrated and covered with EUKITT mounting media (O. Kindler GmbH, Bobingen, Germany). Alizarin Red S staining was used to identify the efficiency of osteogenesis in this study. Sections were stained in Alizarin Red S solution (pH 9, Morphisto, Frankfurt, Germany), re-stained in Alizarin Red S solution (pH 7, Morphisto, Frankfurt, Germany), then dehydrated and mounted in synthetic resin (O. Kindler GmbH, Bobingen, Germany).

To observe the chondrogenic or osteogenic response within the muscle tissue samples, Rabbit polyclonal anti-ACAN (1:150, orb213537) and anti-OCN (1:100, orb259644) antibodies (Biorbyt, Eching, Germany) were utilized for IHC staining. Rabbit-on-Rodent HRP-Polymer (ZYTOMED SYS-TEMS GmbH) was applied as a secondary antibody. Negative control was also set up using Antibody Diluent (ZYTOMED SYSTEMS GmbH, Berlin, Germany) instead of the primary antibodies. Finally, a Vina-Green™ chromogenic kit (Biocare-Medical, Concord, CA, United States) was used to show positive interactions between antigen and antibody.

### 4.5 Histomorphometric analysis

Histological and IHC stainings were captured using the PreciPoint M8 Digital Microscope & Scanner (PreciPoint GmbH, Freising, Germany). The images were histomorphometrically analyzed by the Image-Pro plus 6.0 software (Media Cybernetics, Inc. Silver spring, MD United States). The ratio of the positive-range optical density value (IOD) to the whole range of the sample was the raw staining result.

### 4.6 Statistical analysis

**FIGURE 9:**
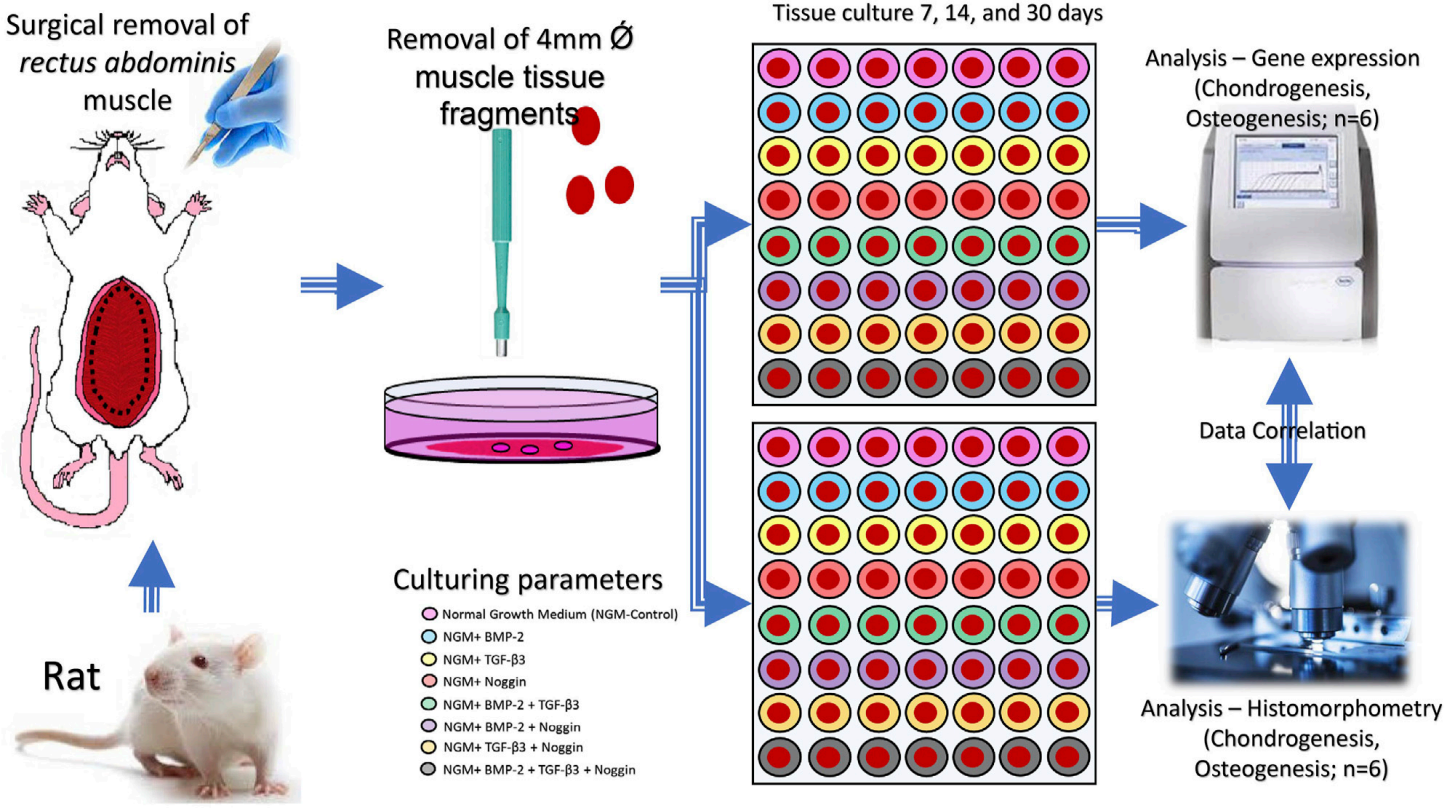
A graphical abstract of the whole experiment.

GraphPad Prism software 8 (La Jolla, CA, United States, http://www.graphpad.com) was used for statistical assessment. Quantile-quantile (q-q) plot was used to test the normality of the data distribution (Supplementary Figure S1–S3). The comparison between different experimental and corresponding control groups was performed by one-way analysis of variance (ANOVA) with Dunnet’s multiple comparisons test. The comparison between each group at different time periods was performed by one-way ANOVA with Tukey’s multiple comparisons test. A significance level of *p* < 0.05 was considered statistically significant. The results are shown as box plots showing the mean and the upper and lower interquartile range with whiskers encompassing the minimum and the maximum value of each group. Rstudio (R-Studio, Boston, MA, United States; http://www.rstudio.com) was utilized to create the final heat maps. Depending on the culture conditions, the heat map was grouped into 8 clusters. The materials and methods were summarized as a graphical abstract in Figure 9.

## 5 Conclusion

Tissue morphogenesis is a tightly modulated temporal and spatial combination of various signaling cues that are improperly elucidated, causing clinical TE processes to fail. Continuing our systematic studies that attempt to understand how the interactions of multiple growth factors regulate osteo-chondrogenesis of muscle tissue over a specific time frame, we have observed clear differences. The combination of BMP-2+TGF-β3, while able to synergize with each other to stimulate osteo-chondrogenesis, also showed that they could antagonize each other in a time-dependent manner. However, the Noggin results were most intriguing. Not only does Noggin appear to be able to antagonize TGF-β3, albeit only at specific temporal intervals, but Noggin appears to be able to synergize with TGF-β3 to promote osteo-chondrogenesis in a temporal manner. This study thus demonstrated a clear need to reconsider the temporal function of growth factors and their inhibitors during the differentiation process in order to achieve more effective TE approaches in clinical applications.

## Data Availability

The datasets presented in this study can be found in online repositories. The names of the repository/repositories and accession number(s) can be found in the article/Supplementary Material.

## Glossary

3D: 3-dimension
Abi3bp: ABI family member 3 binding protein
ACAN: Aggrecan
Actb: Actin beta
Alp: Alkaline phosphatase
ANOVA: Analysis of variance
BMP-2: Bone morphogenetic protein-2
BMSCs: Bone marrow derived mesenchymal stem cells
Col2a1: Collagen type II alpha 1
Col1a1: Collagen type I alpha 1
CNRQs: Calibrated normalized relative quantities
ECM: Extracellular matrix
FBS: Fetal bovine Serum
Gapdh: Glyceraldehyde-3-phosphate dehydrogenase
IOD: Optical density value
IHC: Immunohistochemistry
LMU: Ludwig Maximillian University of Munich
MIQE: Minimum information for publication of quantitative real-time PCR experiments
OCN: Osteocalcin
Polr2e: RNA polymerase II subunit e
P/S: Penicillin and streptomycin
Runx2: Runx family transcription factor 2
Rplp0: Ribosomal protein lateral stalk subunit p0
SEM: Standard error of mean
Sdha: Succinate dehydrogenase complex flavoprotein subunit A
Six1: Sineoculis homeobox homolog 1
Sox9: Sex determining region Y (SRY)-box transcription factor 9
TGF-β3: Transforming growth factor-beta 3
TE: Tissue engineering
RT-qPCR: Quantitative reverse transcription polymerase chain reaction
