# Supplementary material for: Osteochondrogenesis by TGF-β3, BMP-2 and noggin growth factor combinations in an ex vivo muscle tissue model: Temporal function changes affecting tissue morphogenesis
Source: Front Bioeng Biotechnol. 2023 Mar 16;11:1140118. doi: 10.3389/fbioe.2023.1140118 (PMC10060664; doi:10.3389/fbioe.2023.1140118)
Supplement: Supplementary file 1 [file DataSheet2.PDF]

Supplementary Material

Osteochondrogenesis by TGF-β3, BMP-2 and Noggin growth factor combinations in an ex vivo muscle tissue model: temporal function changes affecting tissue morphogenesis

Heng Liu[1,2]*, Peter E. Müller[1], Attila Aszódi[1] and Roland M. Klar[1,3]*

*** Correspondence:** Corresponding Author: email@uni.edu

[1] Department of Orthopaedics and Trauma Surgery, Musculoskeletal University Center Munich (MUM), University Hospital, LMU Munich, Marchioninistr. 15, 81377, Munich, Germany.

[2] Department of Orthopaedics and Traumatology, Beijing Jishuitan Hospital, the Fourth Medical College of Peking University, Beijing, 100035, China.

[3] Department of Oral and Craniofacial Sciences, University of Missouri-Kansas City, School of Dentistry,650 East 25th Street, Kansas City, MO, 634108, USA

*** Correspondence:**
Heng Liu, MD. Email: liuxiaoheng2018@gmail.com;

Roland M. Klar, PhD. Email: rkyh7@umkc.edu

The supplementary material has been uploaded to the Fig**share** with lower information:

DOI: 10.6084/m9.figshare.21836382

LINK: https://doi.org/10.6084/m9.figshare.21836382

Figure Captions

Figure S1: Quantile-quantile (q-q) plot of chondrogenic genes analyses. **(A)** *Col2a1*, **(B)** *Sox9*, **(C)** *Acan*, **(D)** *Six1* and **(E)** *Abi3bp* at 7, 14, and 30 days;

Figure S2: Quantile-quantile (q-q) plot of osteogenic genes analyses. **(A)** *Alp*, **(B)** *Runx2*, **(C)** *Bmp-2*, **(D)** *Ocn*, and **(E)** *Col1a1* at 7, 14, and 30 days;

Figure S3: Quantile-quantile (q-q) plot of histomorphometrical analyses; **A.** Alcian Blue staining. **B.** Alizarin Red S staining. **C.** IHC-ACAN staining. **D.** IHC-OCN staining.

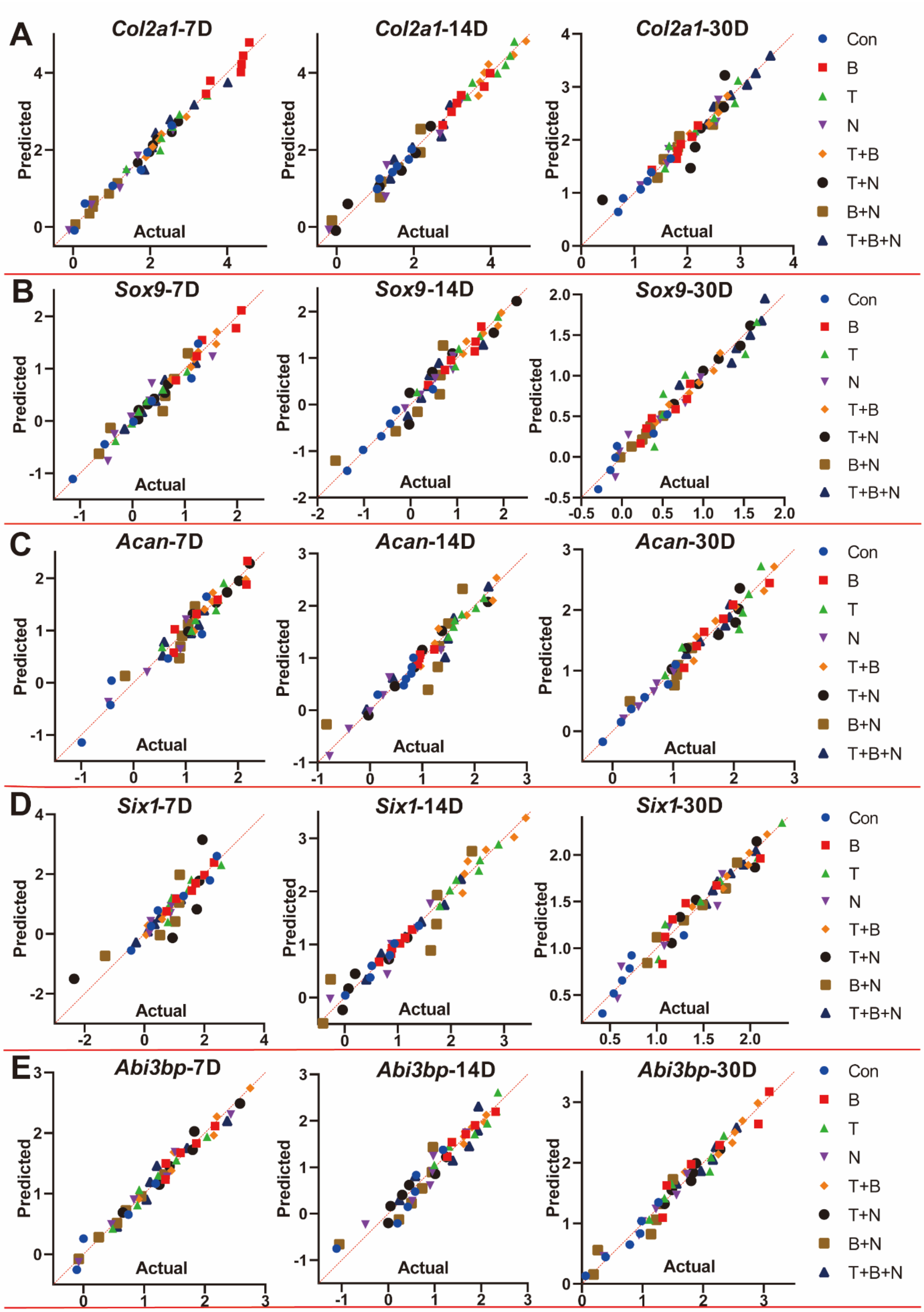
A
Col2a1-7D
Col2a1-14D
Col2a1-30D
B
Sox9-7D
Sox9-14D
Sox9-30D
C
Acan-7D
Acan-14D
Acan-30D
D
Six1-7D
Six1-14D
Six1-30D
E
Abi3bp-7D
Abi3bp-14D
Abi3bp-30D
Predicted
Actual
Con
B
T
N
T+B
T+N
B+N
T+B+N

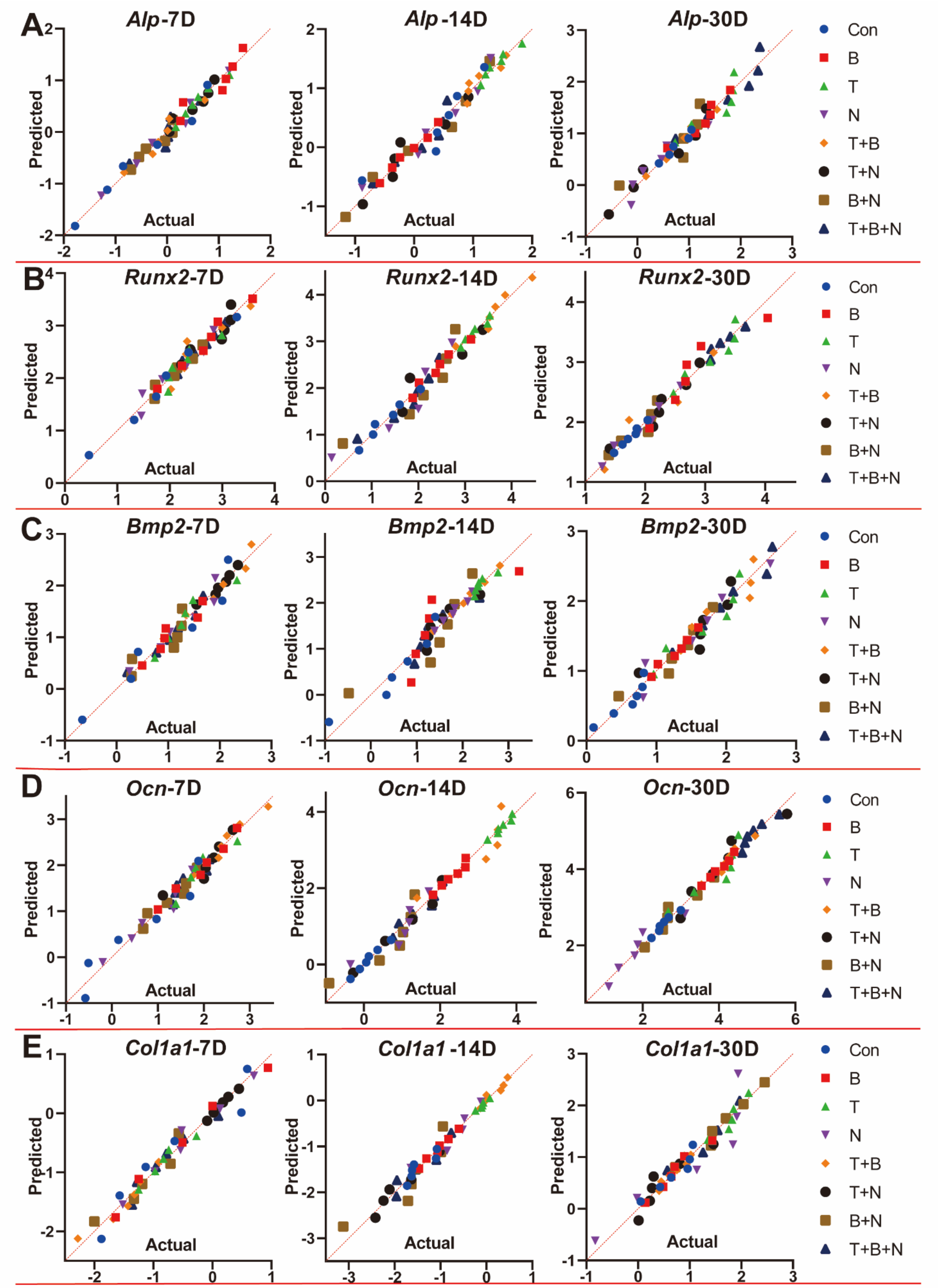
A
Alp-7D
Alp-14D
Alp-30D
B
Runx2-7D
Runx2-14D
Runx2-30D
C
Bmp2-7D
Bmp2-14D
Bmp2-30D
D
Ocn-7D
Ocn-14D
Ocn-30D
E
Col1a1-7D
Col1a1-14D
Col1a1-30D
Predicted
Actual
Con
B
T
N
T+B
T+N
B+N
T+B+N

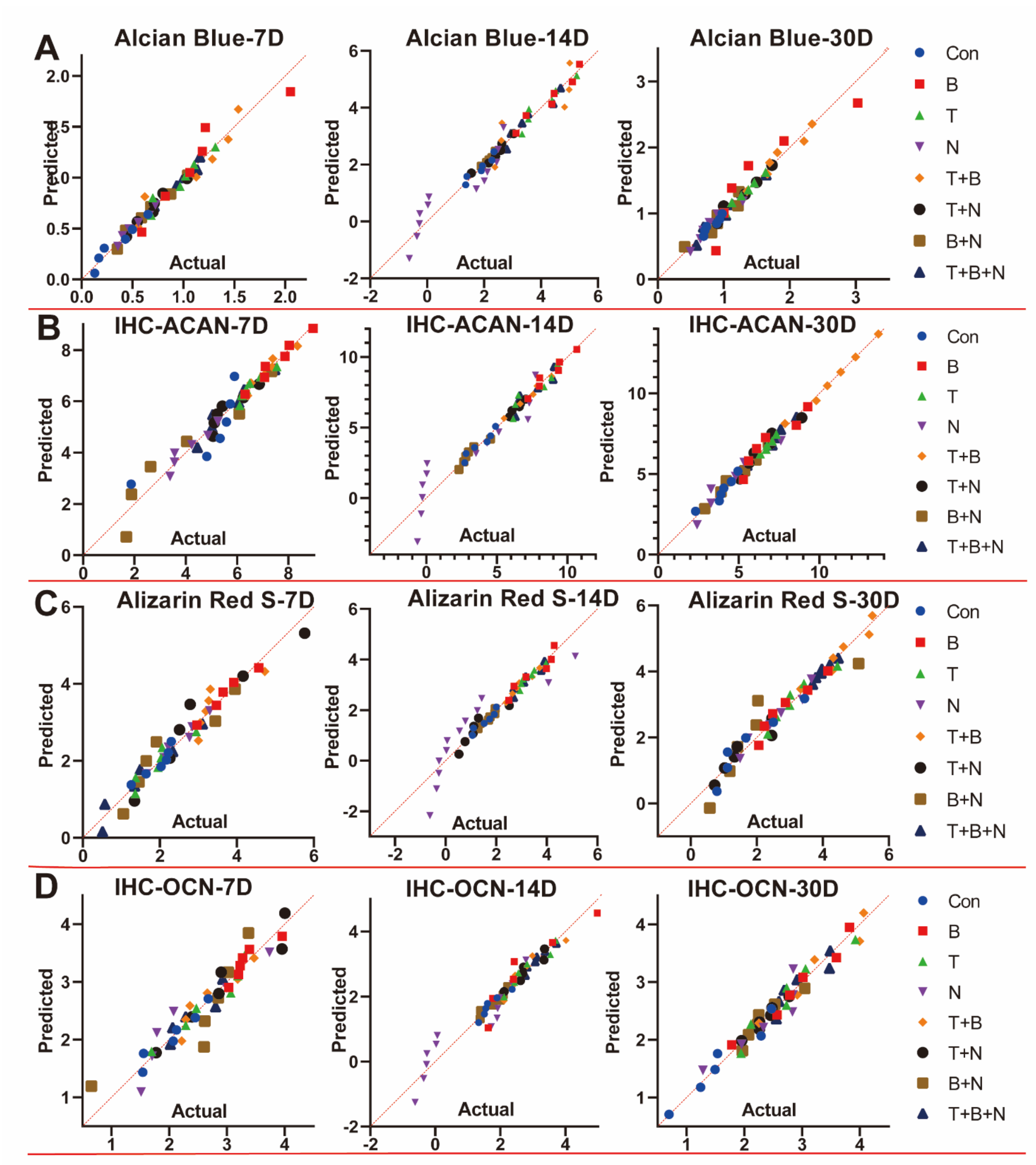
A
Alcian Blue-7D
Alcian Blue-14D
Alcian Blue-30D
B
IHC-ACAN-7D
IHC-ACAN-14D
IHC-ACAN-30D
C
Alizarin Red S-7D
Alizarin Red S-14D
Alizarin Red S-30D
D
IHC-OCN-7D
IHC-OCN-14D
IHC-OCN-30D
Predicted
Actual
Con
B
T
N
T+B
T+N
B+N
T+B+N

Table Captions

Table S1: Statistical analyses of *Col2a1*, *Sox9*, *Acan*, and *Six1* at 7, 14, and 30 days;

Table S2: Statistical analyses of *Abi3bp*, *Alp*, and *Runx2* at 7, 14, and 30 days;

Table S3: Statistical analyses of *Bmp2*, *Ocn*, and *Col1a1* at 7, 14, and 30 days;

Table S4: Statistical analyses of histomorphometrical results at 7, 14, and 30 days.

Table S1. Statistical analyses of *Col2a1, Sox9, Acan and Six1* at 7, 14, and 30 days.

Groups	Time(days)	Target genes			
		Col2a1	***Sox9***	***Acan***	***Six1***
Control	7	1.27±0.40	0.19±0.38	0.25±0.41	1.03±0.47
	14	1.50±0.152	-0.54±0.25	0.65±0.10	0.69±0.19
	30	1.15±0.15	0.06±0.13	0.46±0.18	0.72±0.12
	7 vs.14 P-value	0.8141	**0.0356**	0.5576	0.7264
	7 vs. 30 P-value	0.9381	0.5378	0.8460	0.7562
	14vs.30 P-value	0.6142	0.2064	0.8739	0.9986
BMP-2	7	4.12±0.19	1.45±0.20	1.45±0.25	1.57±0.24
	14	3.31±0.19	1.05±0.19	0.85±0.15	0.98±0.09
	30	1.85±0.12	0.53±0.11	1.74±0.20	1.39±0.17
	7 vs. 14 P-value	**0.0149**	0.2483	0.1402	0.0752
	7 vs. 30 P-value	**<0.0001**	**0.0044**	0.5939	0.7674
	14vs. 30 P-value	**<0.0001**	0.1111	**0.0223**	0.2453
TGF-β3	7	2.46±0.28	0.29±0.19	1.29±0.18	1.35±0.28
	14	4.09±0.21	1.08±0.23	1.61±0.15	2.31±0.17
	30	2.29±0.24	0.89±0.22	1.82±0.26	1.61±0.21
	7 vs. 14 P-value	**0.0008**	0.0553	0.5341	**0.0238**
	7 vs. 30 P-value	0.8856	0.1570	0.1969	0.6971
	14vs. 30 P-value	**0.0003**	0.8299	0.7504	0.1106
Noggin	7	1.21±0.38	0.23±0.29	0.42±0.22	0.81±0.23
	14	1.59±0.49	0.47±0.16	0.13±0.29	0.87±0.26
	30	2.03±0.18	0.36±0.18	0.59±0.11	1.13±0.19
	7 vs. 14 P-value	0.7547	0.7184	0.7352	0.9775
	7 vs. 30 P-value	0.3025	0.9075	0.8936	0.5996
	14vs. 30 P-value	0.6996	0.9305	0.3925	0.7220
TGF-β3 + BMP-2	7	2.34±0.15	1.25±0.13	1.48±0.14	0.58±0.18
	14	4.11±0.20	1.61±0.15	1.69±0.24	2.68±0.20
	30	2.21±0.18	0.85±0.12	1.94±0.23	1.83±0.11
	7 vs. 14 P-value	**<0.0001**	0.1894	0.7720	**<0.0001**
	7 vs. 30 P-value	0.8838	0.1368	0.3119	**0.0004**
	14vs. 30 P-value	**<0.0001**	**0.0039**	0.6943	**0.0095**

TGF-β3 + Noggin	7	2.2±0.15	0.37±0.09	1.63±0.19	1.81±0.19
	14	1.258±0.39	0.89±0.39	0.99±0.32	0.72±0.33
	30	2.04±0.35	1.13±0.14	1.69±0.19	1.6±0.16
	7 vs. 14 P-value	0.1262	0.3179	0.1830	**0.0174**
	7 vs. 30 P-value	0.9360	0.1075	0.9835	0.8176
	14vs. 30 P-value	0.2233	0.7801	0.1371	**0.0466**
BMP-2 + Noggin	7	0.61±0.16	0.34±0.28	0.79±0.19	1.06±0.12
	14	1.35±0.86	0.03±0.37	1.02±0.38	1.13±0.47
	30	1.96±0.19	0.25±0.07	1.01±0.15	1.38±0.16
	7 vs. 14 P-value	0.1198	0.7130	0.8109	0.9820
	7 vs. 30 P-value	**0.0042**	0.9742	0.8345	0.7339
	14vs. 30 P-value	0.2252	0.8353	0.9989	0.8348
TGF-β3 + BMP-2 + Noggin	7	2.62±0.33	0.48±0.18	1.03±0.14	0.44±0.21
	14	2.21±0.28	0.52±0.22	1.19±0.34	1.29±0.27
	30	2.95±0.19	1.42±0.15	1.69±0.12	1.76±0.08
	7 vs. 14 P-value	0.5599	0.9891	0.8703	**0.0291**
	7 vs. 30 P-value	0.6841	**0.0094**	0.1409	**0.0012**
	14vs. 30 P-value	0.1763	**0.0125**	0.3107	0.2803

A significance level of $p<0.05$ was considered statistically significant, which was **bolded** in the table. *Acan = Aggrecan, Sox9 = Sry-box transcription factor 9, Col2a1 = Collagen type II alpha 1 chain, Six1= Six homeobox 1.*

Table S2. Statistical analyses of *Abi3bp*, *Alp* and *Runx2* at 7, 14, and 30 days.

Groups	Time(days)	Target genes		
		Abi3bp	***Alp***	***Runx2***
Control	7	0.94±0.37	-0.45±0.40	1.85±0.39
	14	0.31±0.32	0.39±0.28	1.32±0.19
	30	0.74±0.17	0.74±0.09	1.76±0.08
	7 vs.14 P-value	0.3215	0.1284	0.3393
	7 vs. 30 P-value	0.8776	**0.0265**	0.9664
	14vs.30 P-value	0.5850	0.6790	0.4680
BMP-2	7	1.75±0.15	0.92±0.21	2.65±0.25
	14	1.62±0.16	-0.09±0.15	2.42±0.18
	30	2.13±0.30	1.28±0.17	2.81±0.27
	7 vs. 14 P-value	0.9158	**0.0029**	0.7674
	7 vs. 30 P-value	0.4581	0.3524	0.8804
	14vs. 30 P-value	0.2663	**0.0002**	0.4812
TGF-β3	7	1.18±0.22	0.61±0.15	2.28±0.16
	14	1.83±0.23	1.41±0.10	3.22±0.10
	30	1.75±0.20	1.51±0.201	3.10±0.18
	7 vs. 14 P-value	0.1272	**0.0066**	**0.0015**
	7 vs. 30 P-value	0.1901	**0.006**	**0.0045**
	14vs. 30 P-value	0.9686	0.8910	0.8498
Noggin	7	1.08±0.36	-0.02±0.36	2.10±0.24
	14	0.75±0.29	0.41±0.32	1.74±0.36
	30	1.13±0.19	0.39±0.23	1.93±0.20
	7 vs. 14 P-value	0.7038	0.5886	0.6350
	7 vs. 30 P-value	0.9930	0.6256	0.9022
	14vs. 30 P-value	0.6356	0.9979	0.8777
TGF-β3 + BMP-2	7	1.82±0.27	-0.08±0.21	2.58±0.23
	14	1.81±0.09	1.14±0.12	3.63±0.21
	30	2.42±0.16	0.82±0.19	2.18±0.29

	7 vs. 14 P-value	0.9990	**0.0005**	**0.0234**
	7 vs. 30 P-value	0.1057	**0.0069**	0.5112
	14vs. 30 P-value	0.0979	0.4060	**0.0025**
TGF-β3 + Noggin	7	1.58±0.26	0.51±0.14	2.83±0.17
	14	0.51±0.20	-0.05±0.26	2.20±0.31
	30	1.77±0.13	0.45±0.30	2.28±0.21
	7 vs. 14 P-value	**0.0065**	0.2717	0.1896
	7 vs. 30 P-value	0.8124	0.9882	0.2492
	14vs. 30 P-value	**0.0019**	0.3353	0.9833
BMP-2 + Noggin	7	0.61±0.21	-0.24±0.14	2.12±0.15
	14	0.39±0.31	0.14±0.39	2.04±0.36
	30	0.94±0.23	0.78±0.23	1.91±0.13
	7 vs. 14 P-value	0.7929	0.5914	0.9646
	7 vs. 30 P-value	0.6475	**0.0453**	0.8004
	14vs. 30 P-value	0.2944	0.2545	0.9222
TGF-β3 + BMP-2 + Noggin	7	1.33±0.25	0.001±0.17	2.58±0.15
	14	1.30±0.29	0.09±0.20	1.79±0.26
	30	1.96±0.18	1.79±0.26	3.27±0.10
	7 vs. 14 P-value	0.9969	0.9513	**0.0181**
	7 vs. 30 P-value	0.2085	**0.0001**	**0.0390**
	14vs. 30 P-value	0.1849	**0.0002**	**<0.0001**

A significance level of p<0.05 was considered statistically significant, which was **bolded** in the table. *Alp* = Alka-line phosphatase, *Abi3bp = Abi family member-3 binding protein, Runx2 = Runx family transcription factor 2.*

Table S3. Statistical analyses of *Bmp2*, *Ocn* and *Col1a1* at 7, 14 and 30 days.

Groups	Time(days)	Target genes		
		Bmp2	***Ocn***	***Col1a1***
Control	7	0.95±0.45	0.6±0.44	-0.69±0.42
	14	0.55±0.33	0.133±0.15	-1.45±0.12
	30	0.58±0.11	2.56±0.11	0.69±0.17
	7 vs.14 P-value	0.6796	0.4734	0.1467
	7 vs. 30 P-value	0.7175	**0.0004**	**0.0070**
	14vs.30 P-value	0.9978	**<0.0001**	**0.0001**
BMP-2	7	1.07±0.18	1.92±0.26	-0.49±0.37
	14	1.47±0.35	2.3±0.14	-1.05±0.13
	30	1.26±0.10	4.0±0.13	0.72±0.18
	7 vs. 14 P-value	0.4824	0.3387	0.2887
	7 vs. 30 P-value	0.8427	**<0.0001**	**0.0097**
	14vs. 30 P-value	0.8118	**<0.0001**	**0.0004**
TGF-β3	7	1.36±0.22	1.84±0.2	-0.84±0.13
	14	2.41±0.07	3.61±0.09	-0.08±0.04
	30	1.67±0.21	3.89±0.29	1.64±0.18
	7 vs. 14 P-value	**0.0028**	**<0.0001**	**0.0028**
	7 vs. 30 P-value	0.4509	**<0.0001**	**<0.0001**
	14vs. 30 P-value	**0.0330**	0.6276	**<0.0001**
Noggin	7	1.24±0.26	0.89±0.29	-0.46±0.32
	14	1.82±0.13	0.95±0.28	-0.75±0.21
	30	1.58±0.28	1.87±0.28	0.99±0.47
	7 vs. 14 P-value	0.2284	0.9867	0.8331
	7 vs. 30 P-value	0.5880	0.0698	**0.0280**
	14vs. 30 P-value	0.7527	0.0927	**0.0089**
TGF-β3 + BMP-2	7	1.89±0.27	2.52±0.22	-1.47±0.19
	14	2.1±0.21	2.94±0.35	0.16±0.09
	30	1.94±0.19	4.23±0.18	0.70±0.10
	7 vs. 14 P-value	0.7835	0.5150	**<0.0001**
	7 vs. 30 P-value	0.9819	**0.0010**	**<0.0001**
	14vs. 30 P-value	0.8776	**0.0096**	**0.0398**

TGF-β3 + Noggin	7	2.02±0.11	2.06±0.21	0.15±0.08
	14	1.57±0.18	0.99±0.35	-1.83±0.21
	30	1.62±0.19	4.07±0.40	0.51±0.22
	7 vs. 14 P-value	0.1741	0.0952	**<0.0001**
	7 vs. 30 P-value	0.2518	**0.0018**	0.3506
	14vs. 30 P-value	0.9702	**<0.0001**	**<0.0001**
BMP-2 + Noggin	7	0.90±0.19	1.28±0.20	-1.08±0.22
	14	1.33±0.38	0.67±0.34	-1.65±0.32
	30	1.27±0.18	2.87±0.27	1.62±0.24
	7 vs. 14 P-value	0.5185	0.2930	0.3093
	7 vs. 30 P-value	0.6039	**0.0027**	**<0.0001**
	14vs. 30 P-value	0.9859	**0.0001**	**<0.0001**
TGF-β3 + BMP-2 + Noggin	7	1.07±0.22	1.64±0.14	-0.79±0.22
	14	1.39±0.21	1.44±0.21	-1.39±0.20
	30	2.02±0.22	4.93±0.15	1.42±0.19
	7 vs. 14 P-value	0.5459	0.6957	0.1391
	7 vs. 30 P-value	**0.0189**	**<0.0001**	<**0.0001**
	14vs. 30 P-value	0.1397	**<0.0001**	**<0.0001**

A significance level of p<0.05 was considered statistically significant, which was **bolded** in the table. *Bmp-2 = Bone morphogenetic protein-2, Ocn = Osteocalcin, Col1a1 = Collagen type I alpha 1 chain.*

Table S4. Statistical analyses of histomorphometrical results at 7, 14 and 30 days.

Groups	Time(days)	Staining Medhods			
		**Alcian Blue**	**Alizarin Red S**	**IHC-ACAN**	**IHC-OCN**
Control	7	0.35±0.08	1.94±0.17	4.87±0.62	2.07±0.19
	14	1.87±0.17	1.56±0.16	3.79±0.38	1.71±0.15
	30	0.82±0.05	1.77±0.42	3.93±0.37	1.62±0.27
	7 vs.14 P-value	**<0.0001**	0.6050	0.2680	0.4519
	7 vs. 30 P-value	0.0263	0.9065	0.3593	0.3096
	14vs.30 P-value	**<0.0001**	0.8488	0.9768	0.9561
BMP-2	7	1.15±0.20	3.67±0.22	7.56±0.37	3.35±0.13
	14	4.3±0.35	3.48±0.32	8.79±0.51	2.80±0.52
	30	1.55±0.33	2.89±0.33	6.92±0.67	2.93±0.30
	7 vs. 14 P-value	**<0.0001**	0.8836	0.2609	0.5356
	7 vs. 30 P-value	0.6344	0.1783	0.6827	0.6898
	14vs. 30 P-value	**<0.0001**	0.3632	0.0622	0.9645
TGF-β3	7	0.97±0.10	1.95±0.23	6.60±0.22	2.68±0.26
	14	4.11±0.30	3.31±0.16	7.60±0.57	2.86±0.26
	30	1.31±0.09	3.13±0.30	6.65±0.24	2.76±0.29
	7 vs. 14 P-value	**<0.0001**	**0.0033**	0.1917	0.8825
	7 vs. 30 P-value	0.4214	**0.0092**	0.9955	0.9795
	14vs. 30 P-value	**<0.0001**	0.8648	0.2214	0.9565
Noggin	7	0.52±0.06	2.48±0.24	4.15±0.32	2.30±0.36
	14	2.24±0.15	2.20±0.78	5.87±0.72	2.11±0.16
	30	0.8±0.11	2.56±0.35	4.45±0.77	2.35±0.26
	7 vs. 14 P-value	**<0.0001**	0.9232	0.1689	0.8688
	7 vs. 30 P-value	0.2277	0.9929	0.9363	0.9921
	14vs. 30 P-value	**<0.0001**	0.8746	0.2893	0.8076
TGF-β3 + BMP-2	7	1.09±0.17	3.43±0.26	7.20±0.29	2.70±0.21
	14	3.74±0.54	2.98±0.20	7.66±0.59	2.80±0.27
	30	1.84±0.15	4.58±0.33	10.9±0.82	3.24±0.28
	7 vs. 14 P-value	**0.0002**	0.4939	0.8499	0.9592
	7 vs. 30 P-value	0.2873	**0.0222**	**0.0016**	0.3212
	14vs. 30 P-value	**0.0034**	**0.0022**	**0.0048**	0.4604

TGF-β3 + Noggin	7	0.70±0.08	3.14±0.64	5.66±0.30	2.98±0.36
	14	2.57±0.14	1.22±0.28	6.40±0.19	2.81±0.19
	30	1.30±0.15	1.56±0.29	6.60±0.56	2.37±0.11
	7 vs. 14 P-value	**<0.0001**	**0.0198**	0.3779	0.8647
	7 vs. 30 P-value	**0.0145**	0.0578	0.2226	0.2058
	14vs. 30 P-value	**<0.0001**	0.8446	0.9290	0.4296
BMP-2 + Noggin	7	0.66±0.11	2.24±0.48	3.94±0.95	2.52±0.40
	14	2.21±0.08	1.67±0.11	3.11±0.32	1.81±0.15
	30	0.91±0.12	2.04±0.64	4.88±0.60	2.35±0.16
	7 vs. 14 P-value	**<0.0001**	0.6679	0.6681	0.1601
	7 vs. 30 P-value	0.2385	0.9537	0.5954	0.8873
	14vs. 30 P-value	**<0.0001**	0.8358	0.1859	0.3283
TGF-β3 + BMP-2 + Noggin	7	0.96±0.07	1.56±0.41	5.72±0.45	2.49±0.16
	14	3.63±0.31	3.22±0.21	7.55±0.52	3.15±0.15
	30	1.06±0.16	4.02±0.11	7.04±0.43	2.96±0.17
	7 vs. 14 P-value	**<0.0001**	**0.0020**	**0.0374**	**0.0294**
	7 vs. 30 P-value	0.9442	**<0.0001**	0.1525	0.1373
	14vs. 30 P-value	**<0.0001**	0.1401	0.7244	0.6873

A significance level of p<0.05 was considered statistically significant, which was **bolded** in the table. IHC = Immunohistochemistry, ACAN = Aggrecan, OCN = Osteocalcin.
