# Supplementary figures and images for: Osteochondrogenesis by TGF-β3, BMP-2 and noggin growth factor combinations in an ex vivo muscle tissue model: Temporal function changes affecting tissue morphogenesis
Source: Front Bioeng Biotechnol. 2023 Mar 16;11:1140118. doi: 10.3389/fbioe.2023.1140118 (PMC10060664; doi:10.3389/fbioe.2023.1140118)

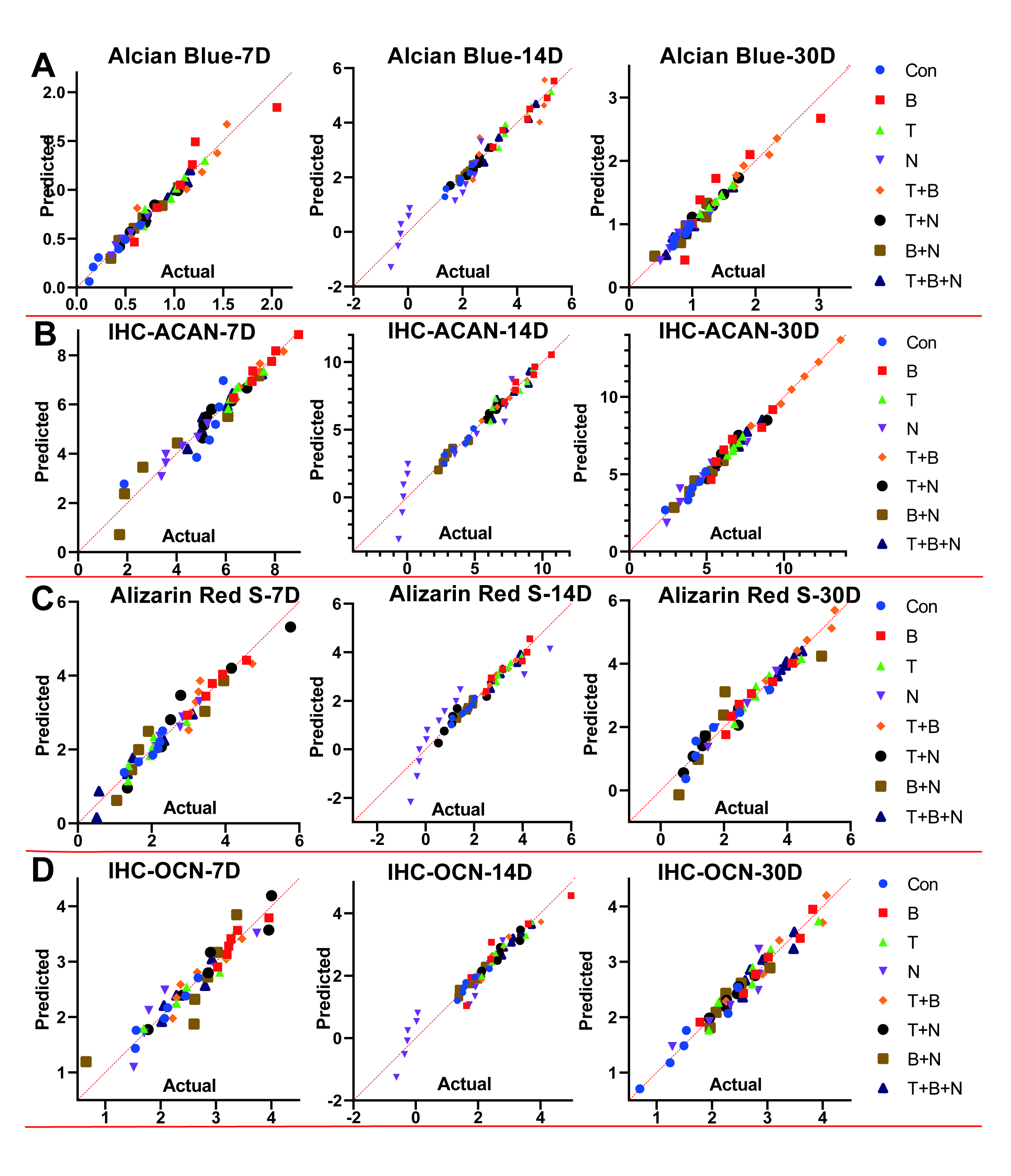

Supplement: Supplementary file 2 [file Image3.TIF]

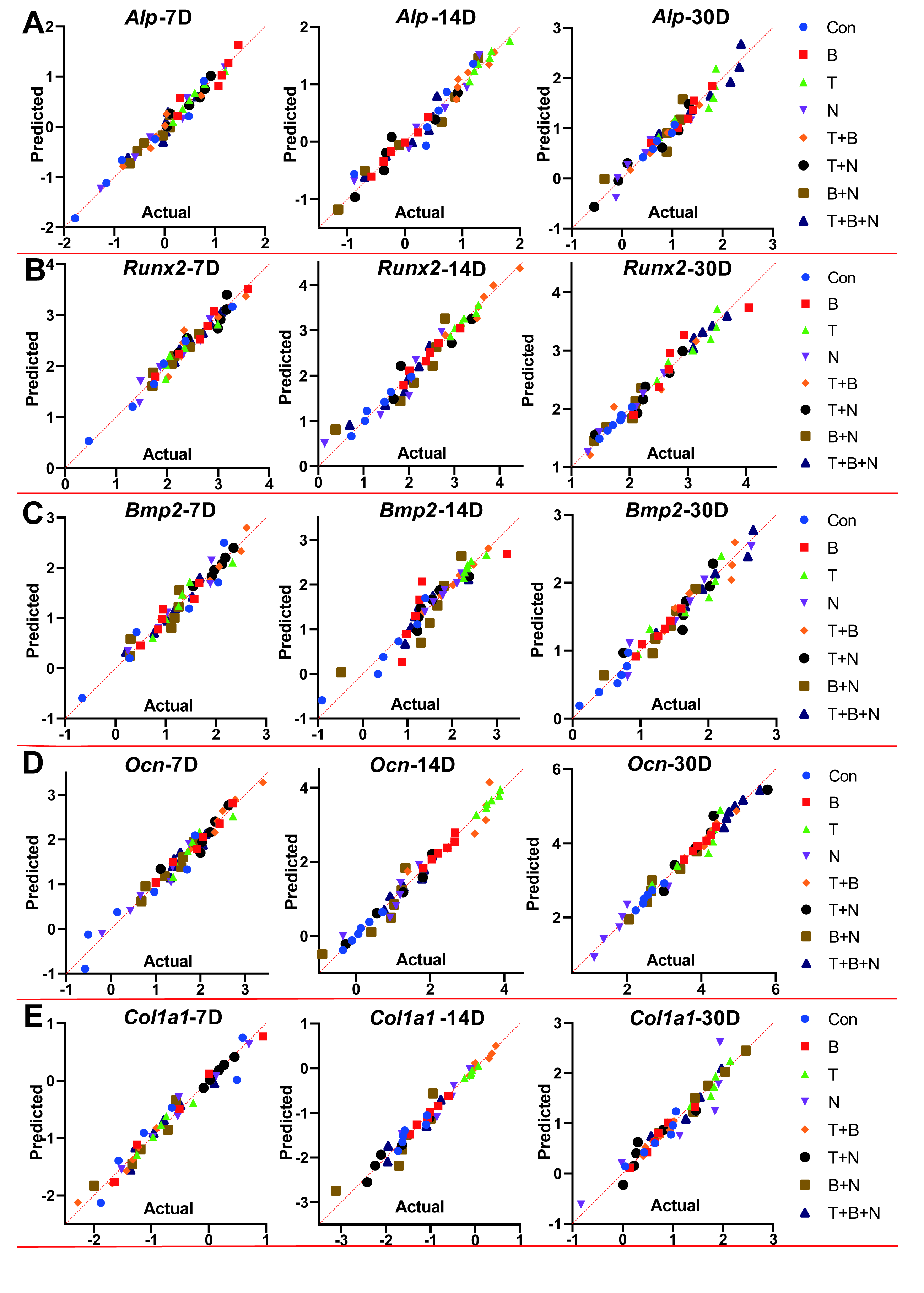

Supplement: Supplementary file 3 [file Image2.TIF]

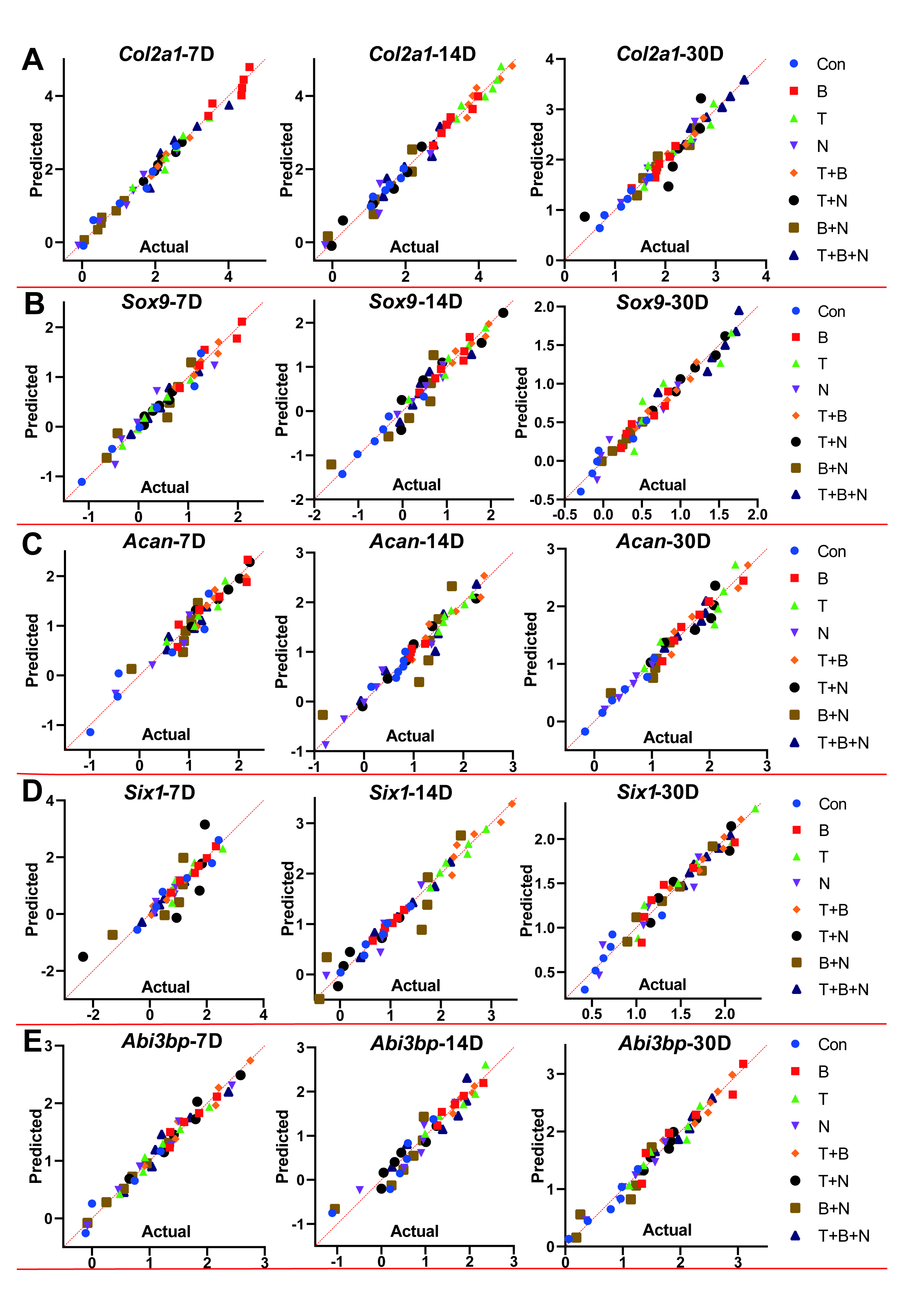

Supplement: Supplementary file 4 [file Image1.TIF]
