# Supplementary material for: Osteochondrogenesis by TGF-β3, BMP-2 and noggin growth factor combinations in an ex vivo muscle tissue model: Temporal function changes affecting tissue morphogenesis
Source: Front Bioeng Biotechnol. 2023 Mar 16;11:1140118. doi: 10.3389/fbioe.2023.1140118 (PMC10060664; doi:10.3389/fbioe.2023.1140118)
Supplement: Supplementary file 5 [file Table2.DOCX]

**Table 1.** The summarized results of/between these three growth factors.

| **Growth Factor Application** | **Effect/Result (Culture period vs reaction intensity chondrogenesis)** | | | **Effect/Result (Culture period vs reaction intensity for osteogenesis)** | | | **Interpretation** |
| --- | --- | --- | --- | --- | --- | --- | --- |
|  | **Day 7** | **Day 14** | **Day 30** | **Day 7** | **Day 14** | **Day 30** |  |
| BMP-2 | **+++** | **++** | **+** | **+++** | **++** | **++** | BMP-2 may function as an initiator only with a short effect period |
| TGF-β3 | **+** | **+++** | **+++** | **+** | **+++** | **+++** | TGF-β3 affects tissue morphogenesis mid-late term |
| Noggin | - | - | - | - | - | - | Noggin inhibits tissue morphogenesis |
| TGF-β3+BMP-2 | - | **+++** | **+++** | - | **++** | **++** | Early stage antagonism that inverts to synergism at later stages |
| TGF-β3+Noggin | **++** | - | **+++** | **+** | - | **++** | Synergistic stimulatory effect at early and late culturing stages with periods of inhibition in between (modulation of tissue morphogenesis?) |
| BMP-2+Noggin | - | - | - | - | - | - | Noggin inhibits BMP-2 function, prevents tissue differentiation |
| TGF-β3+BMP-2 +Noggin | - | **++** | **+++** | - | - | **+++** | Noggin synergizes with BMP-2 only at specific periods and when in the presence of TGF-β3 |

**Table 2.** The target and reference genes information.

|  | Gene name | Accession Nr. | Fwd. (5´-3´) | Rev. (5´-3´) |
| --- | --- | --- | --- | --- |
|  | *Actb* | NM_031144.3 | AGCTATGAGCTGCCTGA | GGCAGTAATCTCCTTCTGC |
|  | *Rplp0* | BC001834.2 | CAACCCAGCTCTGGAGA | CAGCTGGCACCTTATTGG |
| **Reference genes** | *Gapdh* | BC083511.1 | CATGGGTGTGAACCATGA | TGTCATGGATGACCTTGG |
|  | *Polr2e* | BC158787.1 | GACCATCAAGGTGTACTGC | CAGCTCCTGCTGTAGAAAC |
|  | *Sdha* | NM_130428.1 | GCGGTATGAGACCAGTTATT | CCTGGCAAGGTAAACCAG |
|  |  |  |  |  |
|  | *Acan* | NM_022190.1 | CAAGTGGAGCCGTGTTT | TTTAGGTCTTGGAAGCGAG |
|  | *Col2a1* | NM_012929.1 | ATCCAGGGCTCCAATGA | TCTTCTGGAGTGCGGAA |
|  | *Sox9* | NM_080403.1 | CCAGAGAACGCACATCAAG | ATACTGATGTGGCTGGTGG |
|  | *Six1* | NM_053759.1 | CAGGTTCTTGTGGTCGTT | TTTGGGATGGTTGTGAGG |
| **Target genes** | *Abi3bp* | XM_017598145.1 | ACGGGACATTCCTCTCATA | GGTGCCTGAGTTGTCTTT |
|  | *Runx2* | NM_001278484.2 | CCCAAGTGGCCACTTAC | CTGAGGCGGTCAGAGA |
|  | *Alp* | NM_013059.2 | CGACAGCAAGCCCAAG | AGACGCCCATACCATCT |
|  | *Bmp-2* | NM_017178.1 | GGAAGTGGCCCACTTAGA | TCACTAGCAGTGGTCTTACC |
|  | *Ocn* | NM_013414.2 | GCGACTCTGAGTCTGACA | GGCAACACATGCCCTAAA |
|  | *Col1a1* | NM_053304.1 | GGTGACAGAGGCATAAAGG | AGACCGTTGAGTCCATCT |

*Actb = Actin beta, Rplp0 = Ribosomal protein lateral stalk subunit p0, Gapdh = Glyceraldehyde-3-phosphate dehydrogenase, Polr2e = RNA polymerase II subunit e, Sdha = Succinate dehydrogenase complex flavoprotein subunit a; Acan = Aggrecan, Col2a1= Collagen type II alpha 1, Sox9 = Sex determining region Y (SRY)-box transcription factor 9, Six1= Six homeobox 1, Abi3bp = Abi family member 3 binding protein, Runx2 = Runx family transcription factor 2, Alp= Alkaline phosphatase, Bmp-2 = Bone morphogenetic protein-2, Ocn = Osteocalcin, Col1a1 = Collagen type I alpha 1 chain*.
